# OsACL‐A2 negatively regulates cell death and disease resistance in rice

**DOI:** 10.1111/pbi.13058

**Published:** 2019-01-10

**Authors:** Banpu Ruan, Zhihua Hua, Juan Zhao, Bin Zhang, Deyong Ren, Chaolei Liu, Shenglong Yang, Anpeng Zhang, Hongzhen Jiang, Haiping Yu, Jiang Hu, Li Zhu, Guang Chen, Lan Shen, Guojun Dong, Guangheng Zhang, Dali Zeng, Longbiao Guo, Qian Qian, Zhenyu Gao

**Affiliations:** ^1^ State Key Laboratory of Rice Biology China National Rice Research Institute Hangzhou Zhejiang China; ^2^ Department of Environmental and Plant Biology Interdisciplinary Program in Molecular and Cellular Biology Ohio University Athens OH USA

**Keywords:** cell death, disease resistance, *SPL30*, OsACL‐A2, *OsSL*, rice

## Abstract

ATP‐citrate lyases (ACL) play critical roles in tumour cell propagation, foetal development and growth, and histone acetylation in human and animals. Here, we report a novel function of ACL in cell death‐mediated pathogen defence responses in rice. Using ethyl methanesulphonate (EMS) mutagenesis and map‐based cloning, we identified an *Oryza sativa ACL‐A2* mutant allele, termed *spotted leaf 30‐1* (*spl30‐1*), in which an A‐to‐T transversion converts an Asn at position 343 to a Tyr (N343Y), causing a recessive mutation that led to a lesion mimic phenotype. Compared to wild‐type plants, *spl30‐1* significantly reduces ACL enzymatic activity, accumulates high reactive oxygen species and increases degradation rate of nuclear deoxyribonucleic acids. CRISPR/Cas9‐mediated insertion/deletion mutation analysis and complementation assay confirmed that the phenotype of *spl30‐1* resulted from the defective function of OsACL‐A2 protein. We further biochemically identified that the N343Y mutation caused a significant degradation of SPL30^N343Y^ in a ubiquitin‐26S proteasome system (UPS)‐dependent manner without alteration in transcripts of *OsACL‐A2* in *spl30‐1*. Transcriptome analysis identified a number of up‐regulated genes associated with pathogen defence responses in recessive mutants of *OsACL‐A2,* implying its role in innate immunity. Suppressor mutant screen suggested that *OsSL*, which encodes a P450 monooxygenase protein, acted as a downstream key regulator in *spl30‐1*‐mediated pathogen defence responses. Taken together, our study discovered a novel role of OsACL‐A2 in negatively regulating innate immune responses in rice.

## Introduction

Cell death can occur through both apoptotic and non‐apoptotic programmed cell death (PCD) pathways in multicellular organisms (Kutscher and Shaham, 2017; Vaux and Korsmeyer, 1999). Plants have developed hypersensitive responses (HR), which are characterized by PCD of cells around the infection site, to protect them from pathogen attack (Hammond and Parker, 2003; Hofius *et al*., 2007). To better understand the roles of HR cell death in immune responses, many lesion mimic mutants (*LMMs*) with spontaneously induced cell death phenotypes have been investigated in a number of plants, including *Arabidopsis thaliana* (Lorrain *et al*., 2003), groundnut (Badigannavar *et al*., 2002), maize (Walbot *et al*., 1983), wheat (Nair and Tomar, 2001), barley (Wolter *et al*., 1993) and rice (Yin *et al*., 2000). Some *LMMs* developed random lesion and activated immune responses (Staskawicz *et al*., 1995), thus significantly enhancing resistance to disease (Wu *et al*., 2008).

By far, a number of *LMM* genes have been identified to encode proteins with different functions, such as a Cullin domain protein (Liu *et al*., 2017), eukaryotic translation elongation factor 1 alpha (eEF1A)‐like protein (Wang *et al*., 2017), ferredoxin‐dependent glutamate synthase (Sun *et al*., 2017), AAA‐type ATPase (Zhu *et al*., 2016), putative MAPKKK (Wang *et al*., 2015), UDP‐*N*‐acetylglucosamine pyrophosphorylase 1 (Wang *et al*., 2015), splicing factor 3b subunit 3 (Chen *et al*., 2012), a clathrin‐associated adaptor protein (Qiao *et al*., 2010), and an E3 ubiquitin ligase (Zeng *et al*., 2004). These findings indicate that numerous proteins are involved in the regulation of HR cell death and disease resistance.

ATP‐citrate lyase (ACL) is mainly a cytosolic enzyme that catalyses citrate to generate oxaloacetate and acetyl‐CoA (Aoshima, 2007). The product, acetyl‐CoA, is an intermediate metabolite juxtaposed between catabolic and anabolic processes. As the entry point for the tricarboxylic acid (TCA) cycle, acetyl‐CoA can be considered the switch in the oxidation of carbon derived from the catabolism of fatty acids, carbohydrates and amino acids (Fatland *et al*., 2002). ACL has been reported to play an important role in tumour cell propagation, foetal development and growth, and histone acetylation in human and animals (Beigneux *et al*., 2004; Hatzivassiliou *et al*., 2005; Wellen *et al*., 2009). In human, inhibition of ACL expression and activity by either pharmacological inhibitors or RNAi results in growth‐arrest in tumour cells (Hatzivassiliou *et al*., 2005). ACL activity is required to link growth factor‐induced increases in nutrient metabolism to the regulation of histone acetylation and gene expression (Wellen *et al*., 2009). Several *ACL* genes have been cloned in plants such as lupin (Langlade *et al*., 2002) and *Arabidopsis* (Fatland *et al*., 2002). In *Arabidopsis*,* ACL* is required for normal growth and development, and its overexpression activated the wax, cutin and rubber biosynthetic pathways (Fatland *et al*., 2005; Xing *et al*., 2014). In animals, ACL comprises one polypeptide, whereas in plants, it is composed of two distinct subunits, subunit A and subunit B (Hu *et al*., 2015). However, neither phenotypes caused by mutations in *ACL* gene have been reported in plants nor the gene for ACL has been isolated in rice so far.

In order to study molecular mechanisms underlying cell death and disease resistance in rice, a new *LMM* mutant, designated *spotted leaf 30‐1* (*spl30‐1*), was isolated from ethyl methanesulphonate (EMS) treated *O. sativa japonica* cv. Nipponbare. The mutant developed spotted leaves from the seedling stage to the ripening stage. To identify the gene responsible for the phenotype of *spl30‐1,* we applied a map‐based cloning strategy and identified that it encodes the subunit A of the heteromeric *O. sativa* ATP‐citrate lyase (OsACL‐A2). Mutation of *OsACL‐A2* caused a decrease in ACL amount and its total activity, triggered an accumulation of reactive oxygen species (ROS), and enhanced a resistance to bacterial blight, implying a novel role of *OsACL* in innate immunity response.

## Results

### Identification of the *spl30‐1* mutant

**Figure 1:**
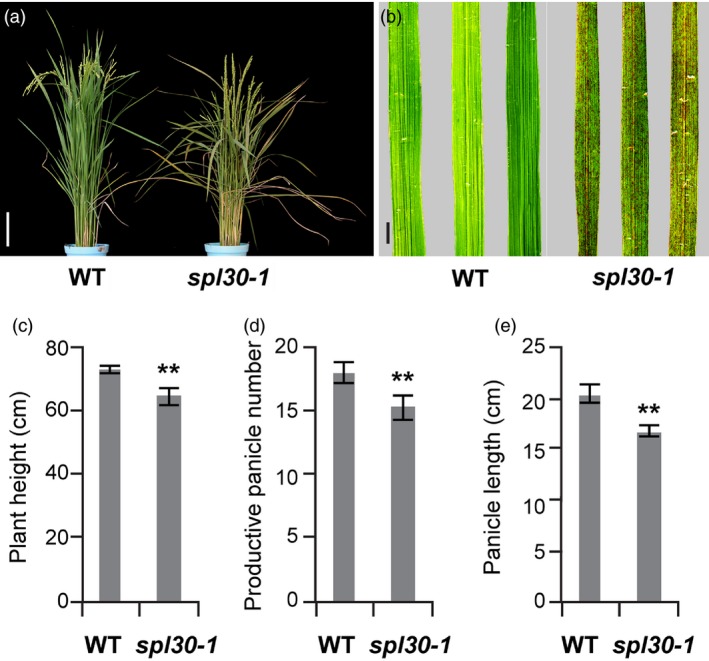
Comparison of phenotype between the wild‐type and *spl30‐1*. (a) Phenotype between wild‐type and *spl30‐1* at the heading stage. Scale bar = 20 cm; (b) Leaf phenotype of wild‐type and *spl30‐1* plants at the heading stage. Scale bar = 2 cm; Comparisons between wild‐type and *spl30‐1* in number of plant height (c), productive panicle (d), and panicle length (e). Error bars means ± SD of 15 independent replicates, ** represent significant difference at 0.01 level by Student's *t*‐test.

The spot leaf mutant *spl30‐1* was isolated from EMS treatment of the *Japonica* rice cultivar Nipponbare. Under field conditions, small and reddish‐brown lesion mimic spots appeared on leaf blades of *spl30‐1* plants from the seedling stage to the ripening stage (Figure 1a,b). At the 3‐leaf seedling stage, no spots appeared on the first fully expanded leaf, whereas numerous spots were found on the second and the third leaf from the top of the tiller (Figure S1A), indicating that the lesion mimic spots are developmentally dependent. In addition, plant height, productive panicle number and panicle length of the *spl30‐1* mutants significantly decreased when compared with wild‐type plants (Figure 1c–e).

### Map‐based cloning of *SPL30*

For genetic analysis of the *spl30‐1* mutant, we crossed the mutant with the wild‐type Nipponbare. All F_1_ plants showed the wild‐type phenotype, suggesting that the mutation is recessive. Among 324 plants examined in the F_2_ population, 241 of them grew as normally as wild‐type, whereas the rest showed mutant phenotype. The resulting 3 : 1 (χ^2^ = 0.17) segregation ratio indicated that *spl30‐1* is a recessive mutant controlled by a single nuclear locus.

**Figure 2:**
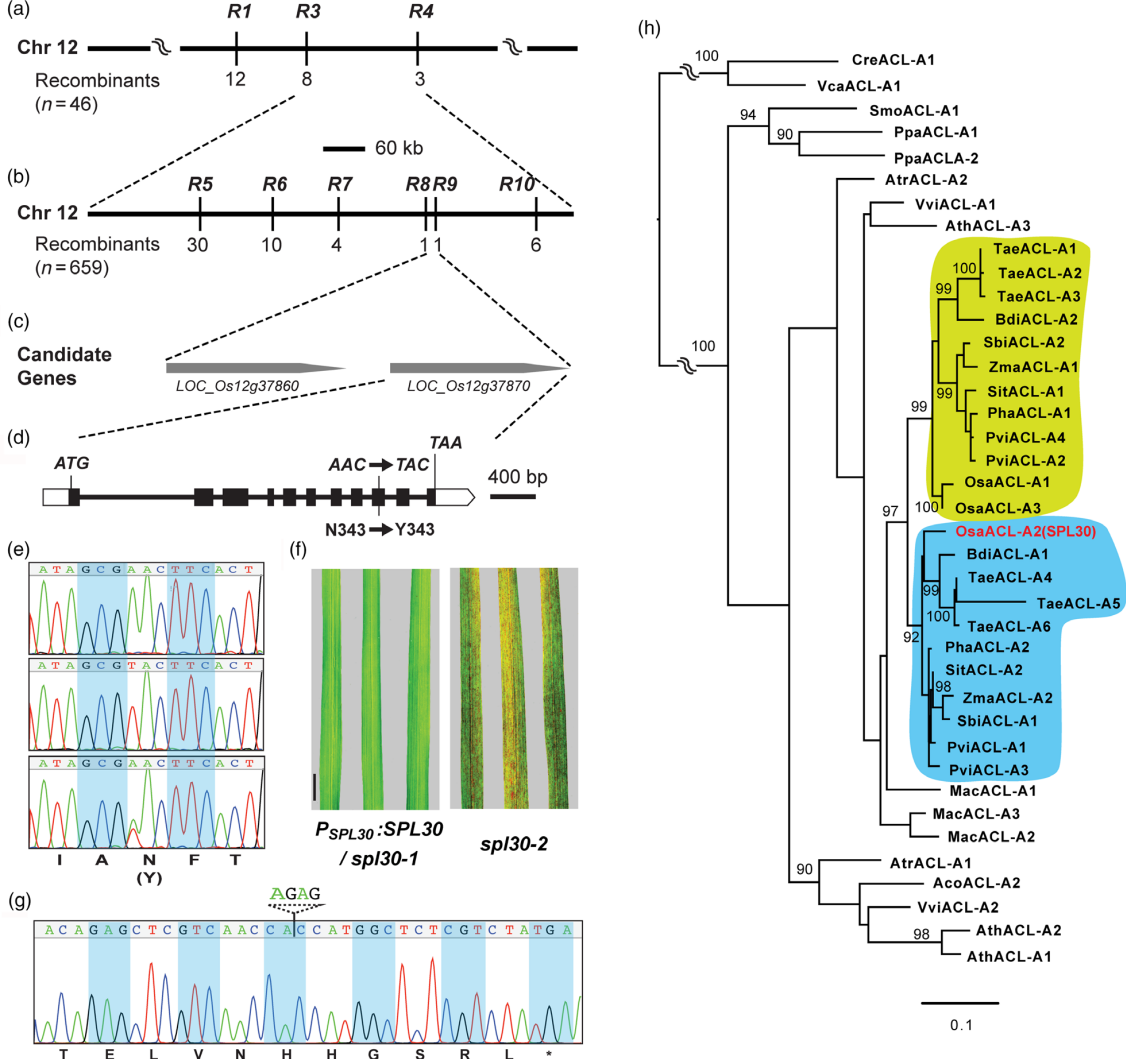
Map‐based cloning and identification of *SPL30*. (a) *SPL30* was preliminarily mapped between markers R3 and R4 on chromosome 12; (b) Fine mapping of *SPL30*. The *SPL30* locus was mapped to a 15.7‐kb region between markers R8 and R9; (c) Two putative open reading frames are located in the 15.7‐kb region; (d) Gene structure of the candidate gene *LOC_Os12g37870*. Black rectangles represent exons. The point mutation of A to T on the ninth exon led to amino acid change in Asn to Tyr. (e) Comparison of sequencing chromatograms of mutated sites in wild‐type (top), *spl30‐1* (middle) and complementation (bottom) plants. (f) Leaf phenotype of complementation lines and *spl30‐2* plants. Scale bar = 4 cm. (g) Sequencing chromatogram of mutated site in *spl30‐2*. (h) Phylogenetic analysis of the *SPL30* family in 17 plants indicates functional divergence of monocotyle ACL‐A subunits of ACL enzymes. The full‐length protein sequences of SPL30 homologous were aligned and used to generate a maximum likelihood tree. Statistic significance equal or greater 90% of 1000 bootstrap re‐samplings are indicated in each node. See Data S1 for the locus identifiers. Species abbreviations are as follows. *Aco*:* Ananas comosus*,* Ath*:* Arabidopsis thaliana*,* Atr*:* Amborella trichopoda, Bdi: Brachypodium distachyon, Cre: Chlamydomonas reinhardtii, Mac: Musa acuminata, Osa: Oryza sativa, Pha: Panicum hallii, Ppa: Physcomitrella patens, Pvi: Panicum virgatum, Sbi: Sorghum bicolor, Sit: Setaria italica, Smo: Selaginella moellendorffii, Tae: Triticum aestivum, Vca: Volvox carteri, Vvi: Vitis vinifera, Zma: Zea mays*.

To genetically map the locus responsible for the mutant phenotype, *spl30‐1* was crossed with an *indica* rice variety, Nanjing 6, and 659 F_2_ plants exhibiting the mutant phenotype were collected. In total, 183 Simple Sequence Repeats (SSRs) and 41 Sequence Tagged Sites (STSs) that are evenly distributed on 12 chromosomes of rice were genotyped. We first mapped the mutant locus between two markers, R3 and R4, on chromosome 12 by analysing 46 F_2_ plants (Figure 2a). Further fine mapping analysis with the total 659 F_2_ mutants allowed us to target the locus within a 15.7‐kb region between R8 and R9 markers (Figure 2b). According to the Rice Genome Annotation Project (RGAP, http://rice.plantbiology.msu.edu), two predicted open reading frames (ORFs), *LOC_Os12g37860* and *LOC_Os12g37870*, were found in this region (Figure 2c). DNA sequencing of these two genes in *spl30‐1* revealed a single nucleotide transversion of A‐to‐T in the ninth exon of *LOC_Os12g37870*, which caused an Asn‐to‐Tyr substitution at the 343th amino acid (Figure 2d,e). Therefore, *LOC_Os12g37870* was selected as the candidate gene.

### Confirmation of *LOC_Os12g37870* responsible for the mutant phenotype of *spl30‐1*

To confirm *SPL30* is *LOC_Os12g37870*, we carried out both complementation and CRISPR/Cas9‐mediated insertion/deletion mutation analyses. First, we cloned 7.5 kb genomic sequence of *LOC_Os12g37870* into a binary vector pCAMBIA1300 and transformed it into *spl30‐1*. In total, 13 independent T_0_ transformants were obtained. Phenotypic analysis confirmed that all of them were able to rescue the *spl30‐1* mutant phenotype by increasing plant height, recovering effective tiller number and eliminating lesion‐mimic spots (Figure S1B; Figure 2f).

We further disrupt *LOC_Os12g37870* using the CRISPR/Cas9 technology. Based on lesion‐mimic phenotype and sequencing results, we obtained two independent deletion alleles, designated *spl30‐2* and *spl30‐3*, which had 4‐bp and 1‐bp deletions, respectively, in the second exon of *LOC_Os12g37870* (Figure S2). Both new deletion mutations led to a reading frame shift after position 137 bp downstream the start codon of *LOC_Os12g37870* and the formation of a premature stop codon, suggesting that the lesion mimic phenotype observed in *spl30‐2* and *spl30‐3* were caused by the functional disruption of *LOC_Os12g37870* (Figure 2f,g, Figure S3). Together with the complementation result of *spl30‐1* by *LOC_Os12g37870*, we concluded that *SPL30* is *LOC_Os12g37870*. Hereafter, we termed this gene *SPL30*.

### Phylogenetic analysis of SPL30

Sequence analysis revealed that SPL30 (OsACL‐A2) is a homolog to the *A. thaliana ATP citrate lyase (ACL)‐A* genes with 79.9%, 78.5% and 87% identity in AtACL‐A1, A2 and A3 deduced amino acid sequences respectively (Figure S4). It spans 3408 bp with 11 exons and the encoded polypeptide is composed of 423 amino acid residues with molecular mass of 45 kDa. There are three and two loci discovered in the Arabidopsis genome to encode ACL‐A and ACL‐B subunits respectively (Fatland *et al*., 2002). This raised a question as to why *spl30‐1/2/3* showed a unique phenotype if multiple genes also encode an ACL‐A subunit in rice. One possibility is that SPL30 has gained a diverged function from its homologous sequences in rice. Since ACL‐A and ACL‐B are clearly distinct in their domain structures, we examined the phylogenetic relationship of *ACL‐A* genes in 17 plant genomes, including 10 monocots, three dicots, two basal land plants and two algae. ACL‐A has been shown to evolve through gene fusion from α‐subunits of succinyl‐CoA synthetase (SCSα) and citrate synthase (CS; Fatland *et al*., 2002), which contain ATP‐grasp 2 and Citrate bind domains respectively. We searched the predicted proteomes by BLASTp (Altschul *et al*., 1990) using the seed sequences of these two domains from Pfam (Version 31, http://pfam.xfam.org) as query. The presence of ATP‐grasp 2 and Citrate bind domains in each hit protein sequence was further verified by hmmscan (http://hmmer.org) against the Pfam‐A profile HMM database (*e*‐value cutoff = 1). In total, 39 ACL‐A homologous sequences were identified (Data S1) and subject to a maximum likelihood phylogenetic analysis (Stamatakis, 2014).

The resulting phylogenetic tree suggested that the land plant *ACL‐A* genes were the descendants of one single ancestral gene homologous to the ancestor of algal *ACL‐A* genes (Figure 2h). While algal species contain one *ACL‐A* gene, most land plants possess two or more members. Interestingly, two distinct clades of *Gramineae Poaceae ACL‐A* genes can be found to form a large and separate cluster from the basal monocotyle plant, *Musa acuminata* and eudicotyle plants, indicating their functional divergence. In addition, *SPL30* (*OsACL‐A2*) discovered in this work resides in a clade that is significantly diverged from its two paralogs, *OsACL‐A1* and *OsACL‐A3*, in rice, suggesting subfunctionalization of *ACL‐A* genes in *Gramineae Poaceae*.

### Reduced ACL activities in *spl30* mutants

**Figure 3:**
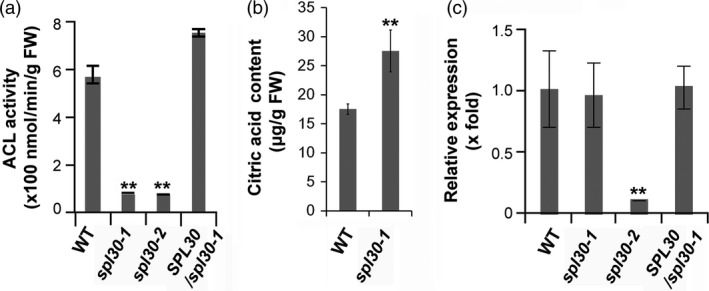
Molecular characteristics of *SPL30*. (a) Enzyme activity of ACL in wild‐type, *spl30‐1*, complementation and *spl30‐2* lines determined in leaves at heading stage. FW, fresh weight. Error bars means ± SD of three biological repeats. ** represent significant difference at 0.01 by Student's *t*‐test. (b) Leaf contents of citric acid in wild‐type, *spl30‐1* and *spl30‐2* plants at heading stage. FW, fresh weight. Error bars means ± SD of three independent replicates, * represent significant difference at 0.05 level by Student's *t*‐test. (c) Quantitative RT‐PCR analysis of *SPL30* expression levels in leaves of wild‐type, *spl30‐1*, complementation and *spl30‐2* lines at heading stage. Error bars means ± SD of three biological repeats. ** represent significant difference at 0.01 by Student's *t*‐test.

We compared the total ACL activity in leaf tissues of wild‐type, *spl30‐1*,* spl30‐2* and complementation lines. Interestingly, the total ACL activities of *spl30‐1* and *spl30‐2* were reduced to approximately 10% and 8%, respectively, of that in wild‐type. In agreement to the phenotypic complementation results, the total ACL activity was comparable between complementation plants and wild‐type (Figure 3a). Since citric acid is a direct substrate of ACL, the decrease in ACL activity will retard its catabolism. As a consequence, the contents of citric acid in *spl30‐1* was significantly higher than that in wild‐type (Figure 3b).

### The UPS‐mediated degradation of SPL30^N343Y^ attenuated ACL activity in *spl30‐1*

**Figure 4:**
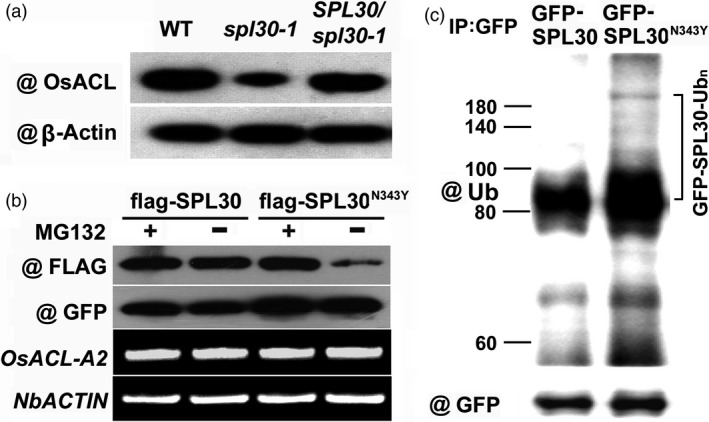
SPL30^N343Y^ is degraded in UPS‐dependent manner. (a) Western blot analysis of OsACL protein levels in leaves of wild‐type, *spl30‐1* and complementation plants at the heading stage. (b) SPL30^N343Y^ degradation in *Nicotiana benthamiana*. SPL30 and SPL30^N343Y^ were fused with the flag tag and coexpressed with GFP in *N. benthamiana* by an agroinfiltration method. The transfected *N. benthamiana* were treated with or without MG132 for 48 h. Total protein was detected with anti‐flag and anti‐GFP antibodies. The expression of *OsACL‐A2* and *NbACTIN* was analysed by RT‐PCR. (c) Ubiquitination of SPL30 and SPL30^N343Y^ proteins *in vivo*. *Nicotiana benthamiana* leaves were transfected by injection of *Agrobacterium* EHA105 cells harbouring 35S:GFP‐SPL30 and 35S:GFP‐SPL30^N343Y^ construct respectively. Total proteins were extracted and immunoprecipitated with GFP‐Trap‐A. Then, they were probed with anti‐Ub and anti‐GFP antibodies respectively. The numbers on the left show the molecular masses of marker proteins in kilodaltons. Bracket indicates poly‐ubiquitinated GFP‐SPL30^N343Y^.

The low ACL activity suggested a defective function of SPL30 in *spl30‐1* and *spl30‐2* (Figure 3a). It is not surprising that truncation of SPL30 caused by a premature stop codon disrupts the total enzymatic activity of ACL in *spl30‐2*. However, the full‐length of SPL30 can be produced in *spl30‐1* except for the change in N343Y (Figure 2d). To dissect the influence of N343Y on the function of SPL30, we first questioned whether the transcript level of *SPL30* had been perturbed in *spl30‐1*. By qRT‐PCR, we found that the transcripts of *SPL30* did not change in *spl30‐1* but reduced fivefold in *spl30‐2* (Figure 3c), suggesting that the function of *SPL30* was not disrupted at the transcriptional level in *spl30‐1*. To monitor the protein abundance of SPL30, we developed a rabbit polyclonal antibody and detected a significant reduction in SPL30^N343Y^ in *spl30‐1*, but not SPL30 in wild‐type plants and the complementation line (Figure 4a), which indicated that the point mutation N343Y enhanced a post‐translational degradation of SPL30.

We then examined whether SPL30^N343Y^ but not SPL30 is a protein substrate of the ubiquitin‐26S proteasome system (UPS) in a heterologous plant expression system by expressing both proteins in leaves of *Nicotiana benthamiana*. After co‐expressing free GFP proteins along with FLAG tagged SPL30^N343Y^ or SPL30 proteins in leaf tissues of *N. benthamiana* for 48 h, we further infiltrated the same area with a 26S proteasome inhibitor MG132 or DMSO (solvent control) and cultured the samples for additional 48 h at 22°C in dark. Here, the expression of free GFP proteins was used to monitor the efficacy of transient protein expression in different infiltration experiments. To further ensure an equal transcription level of *SPL30* or *spl30‐1* in different leaf tissues, the mRNAs of both *NbActin* and *SPL30/spl30‐1* were quantified by RT‐PCR.

As shown in Figure 4b, we found that protein abundance of SPL30^N343Y^ but not SPL30 was dramatically reduced in leaf tissues without MG132 treatment although transcript amount of *spl30‐1* and *SPL30* retained the same. Since a UPS‐mediated protein degradation can be suppressed by MG132, an effective inhibitor that blocks the activities of proteases in the central core particle of the 26S proteasome (Lee and Goldberg, 1996), the abundance of SPL30^N343Y^ would be recovered by the MG132 treatment. Consistent with this notion, we observed that the protein level of SPL30^N343Y^ in leaf tissues treated with MG132 maintained the same level as SPL30 in those tissues treated with or without MG132. In addition, neither the transcripts of *spl30‐1* nor the protein level of GFP did MG132 influence. Then, we detected a smear of bands corresponding to larger molecules, which represents the feature of ubiquitinated forms of proteins in the GFP‐SPL30^N343Y^ sample via immunoprecipitation with anti‐GFP antibody and Western blot analysis with anti‐Ub and anti‐GFP antibodies (Figure 4c). Therefore, SPL30^N343Y^ was suggested a protein substrate targeted for degradation in a UPS‐dependent manner in plants.

### Constitutive expression of *SPL30*

Since acetyl‐CoA produced by ACL is an essential intermediate metabolite, we expected that SPL30 is constitutively expressed in rice. By real‐time quantitative PCR (qRT‐PCR), we found that *SPL30* is widely expressed in various tissues, including roots, culms, leaves, leaf sheaths and panicles, with the highest transcript level detected in leaves and sheaths, whereas the lowest level observed in roots (Figure S5A). We further fused a 2341‐bp genomic sequence upstream of the start codon of *SPL30* with the coding sequence of β‐glucuronidase (GUS) and transformed the fusion gene into wild‐type plants. Histochemical assay demonstrated GUS activity in roots, culms, leaves and panicles in eight T_0_ transgenic plants (Figure S5B–H), further confirming the constitutive expression pattern of *SPL30*.

Then we constructed a p35S::SPL30::GFP vector and transformed it into rice protoplasts and tobacco (*N. benthamiana*) leaf epidermal cells to detect the subcellular localization of SPL30. Under confocal microscope, the fusion protein was observed in nucleus and cytoplasm (Figure S6A,B).

### Reduction in SPL30^N343Y^ led to programmed cell death in *spl30‐1*

**Figure 5:**
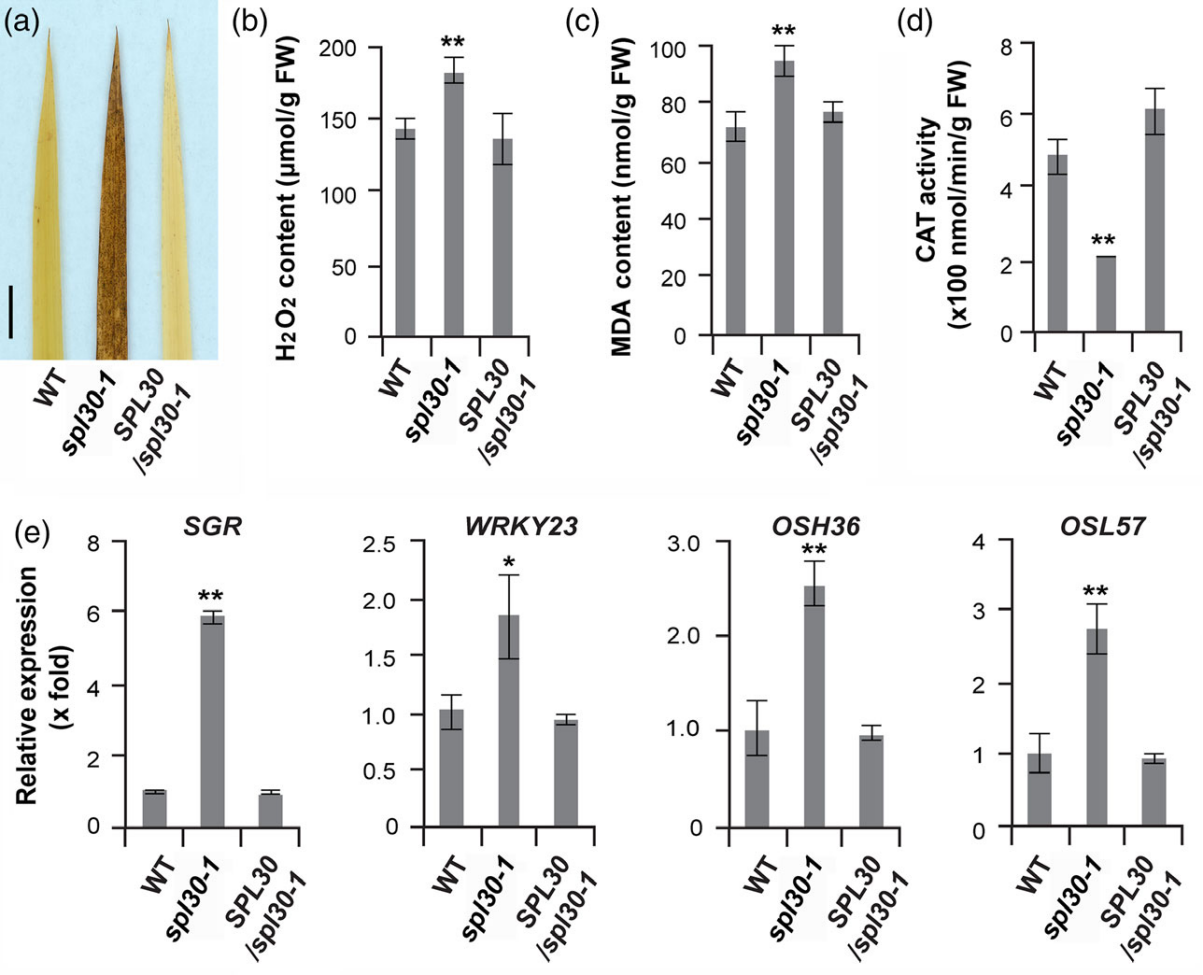
*spl30*‐induced H_2_O_2_ production and up‐regulated senescence‐related genes. (a) DAB assay of H_2_O_2_ in leaf tip tissue of wild‐type, *spl30‐1* and complementation plants at heading stage. (b) H_2_O_2_ contents in leaves tissue of wild‐type, *spl30‐1* and complementation plants at heading stage. FW, fresh weight. (c) malondialdehyde contents in leaves tissue of wild‐type, *spl30‐1* and complementation plants at heading stage. FW, fresh weight. (d) CAT activity in leaves tissue of wild‐type, *spl30‐1* and complementation plants at heading stage. FW, fresh weight. (e) Relative expression levels of senescence‐related genes in wild‐type, *spl30‐1* and complementation plants. Error bars means ± SD of three independent replicates. * and ** represent significant difference at 0.05 and 0.01 level, respectively, by Student's *t*‐test.

The lesion‐mimic spots produced in *spl30‐1* leaves suggested that reduction in SPL30^N343Y^ triggers cell death responses. One typical cell death response is characterized by an over accumulation of hydrogen peroxide (H_2_O_2_) in cells. By incubating leaves of wild‐type, *spl30‐1* and complementation plants in 3, 3′‐diaminobenzidine (DAB) for 8 h, brown coloration was developed on leaves of *spl30‐1* but not those from wild‐type and the complementation plants (Figure 5a), indicating that a significant amount of H_2_O_2_ was accumulated in *spl30‐1*. We further quantified that the concentration of H_2_O_2_ in the mutant is significantly higher than that in both wild‐type and the complementation plants, and so is the production of malondialdehyde (MDA), another biomarker related to cell death and lipid peroxidation (Ayala *et al*., 2014). Further enzymatic analysis demonstrated that the catalase activity was reduced 2.5‐fold in *spl30‐1*, implying its up‐regulation of oxidative stress responses (Figures 5b–d). Consistently, the transcripts of four senescence‐related transcription factors, *SGR*,* WRKY23*,* Osh36* and *Osl57* were detected to be up‐regulated by 6‐, 2‐, 2.5‐ and 2.8‐fold, respectively, in *spl30‐1* (Figure 5e).

To examine cell death in *spl30‐1* at cellular level, we observed the extent of chromatin condensation in mature leave cells, a cellular hallmark of PCD (Simeonova *et al*., 2000). Since chromatin condensation in PCD cells resulted from stress‐induced endonucleolytic degradation of nuclear DNA, their nuclei can be detected by terminal deoxyribonucleotidyl transferase‐mediated dUTP nick‐end labelling (TUNEL). Our results showed that in contrast to few TUNEL‐positive nuclei observed in wild‐type, the majority of nuclei in *spl30‐1* were TUNEL positive (Figure S7). Therefore, reduction in SPL30^N343Y^ led to programmed cell death in *spl30‐1*.

### Enhanced innate immune responses in *spl30‐1* and *spl30‐2*

**Figure 6:**
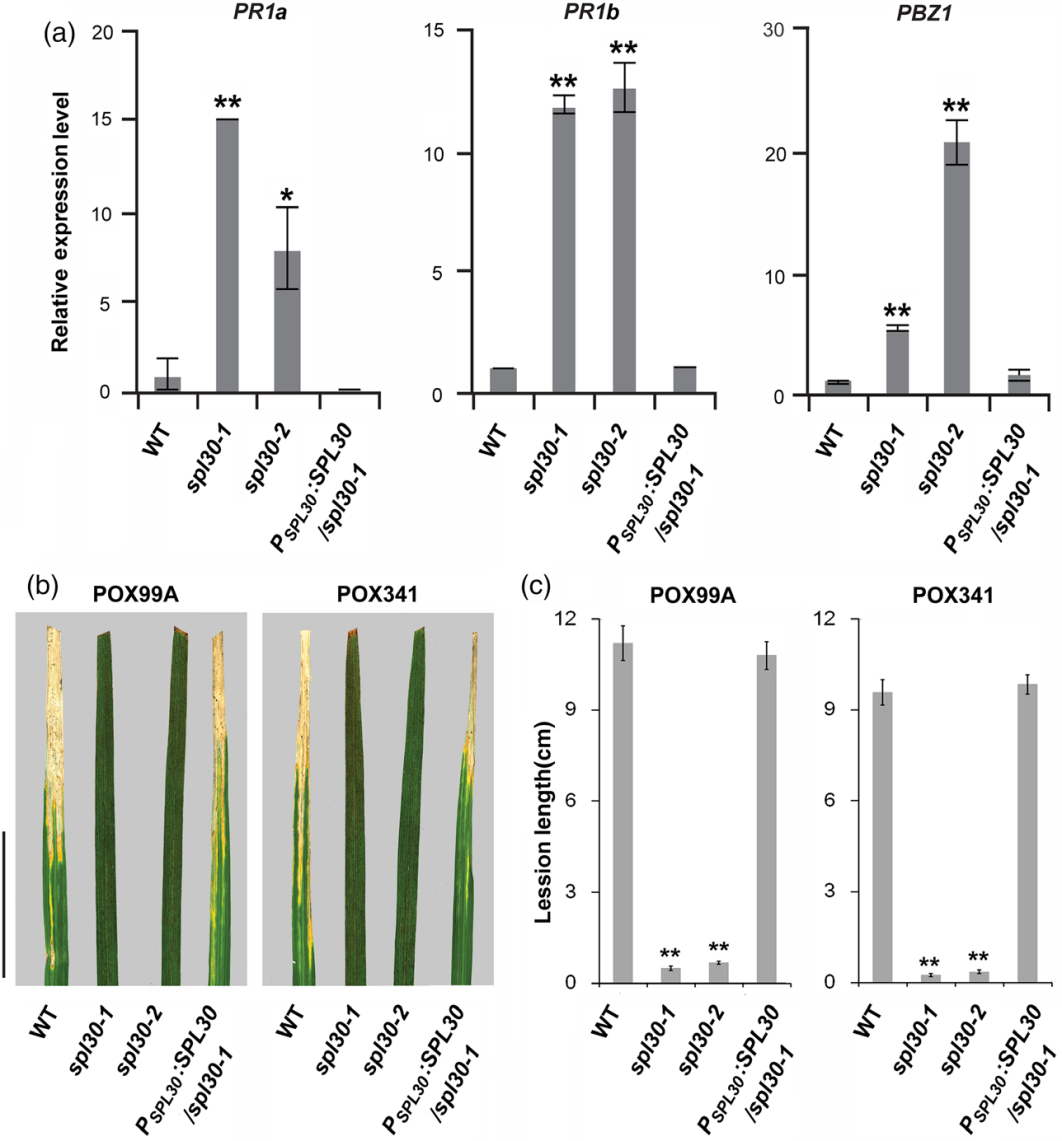
*spl30*‐induced pathogenesis‐related genes and disease resistance. (a) Relative expression levels of pathogenesis‐related genes in wild‐type, *spl30‐1*,* spl30‐2* and complementation plants at heading stage. Error bars means ± SD of three independent replicates. * and ** represent significant difference at 0.05 and 0.01 level, respectively, by Student's *t*‐test. (b) Leaf phenotype of wild‐type, *spl30‐1*,* spl30‐2* and complementation lines (from left to right) after inoculation of plant leaves with bacterial blight pathogen PXO99^A^ (left) and PXO341 (right). Scale bar = 4 cm. (c) Lesion length after inoculation of plant leaves with bacterial blight pathogen PXO99^A^ and PXO341. Error bars means ± SD of ten independent replicates. ** indicate a statistically significant difference at *P* < 0.01 by Student's *t*‐test.

In order to understand the global oxidative stress responses in *spl30‐1*, we performed an RNA‐seq analysis of leave transcriptomes in wild‐type, *spl30‐1* and *spl30‐2* plants at the heading stage. In total, 3060 differentially expressed genes (DEGs; 1444 up‐regulated and 1616 down‐regulated) and 1872 DEGs (1003 up‐regulated and 869 down‐regulated) were identified with >twofold changes [false discovery rate (FDR) < 0.05] of transcripts in *spl30‐1* and *spl30‐2* respectively (Table S2). Consistent with the similar phenotype observed, among these DEGs, 792 and 636 were common to be up‐regulated and down‐regulated, respectively, in *spl30‐1* and *spl30‐2* plants (Figure S8). According to Rice Gene Ontology (GO) annotation, these DEGs could be classified into three categories: cellular component, molecular function and biological process (Figure S9A,B). In respect of biological process, high percentage of the DEGs were found in carbohydrate metabolism, secondary metabolic process and response to biotic stimulus in both *spl30‐1* and *spl30‐2* plants, including two chitinase genes and two WRKY genes (Table S2), which confirmed increased significantly by real‐time PCR in *spl30‐1* and *spl30‐2* compared to wild‐type (Figure S9C). The expression of three pathogenesis‐related genes, *PR1a*,* PR1b* and *PBZ1,* were further confirmed by qRT‐PCR to be up‐regulated in *spl30‐1* and *spl30‐2* plants (Figure 6a).

Considering elevated expression of defence response genes, we challenged wild‐type, *spl30‐1*,* spl30‐2* and complementation plants with bacterial blight (*Xanthomonus oryzea pv. Oryzea*) strains PXO99^A^ and PXO341 at the heading stage by leaf‐cutting inoculation. At 16 days post inoculation (DPI), average leaf lesion lengths, which were caused by pathogen infection through xylem vessels, were measured to quantify the pathogen responses in different plants. The results showed that the average leaf lesion lengths yielded in *spl30‐1* and *spl30‐2* were significantly shorter than those detected in the wild‐type and complementation plants upon the infection of PXO99^A^ and PXO341 (Figure 6b,c), suggesting enhanced innate immune responses in *spl30‐1* and *spl30‐2*.

### *SPL30* is epistatic to *OsSL* in pathogen response pathway

To identify pathways in *SPL30*‐mediated pathogen defence responses, we carried out EMS‐mutagenesis screen for suppressors in *spl30‐1*. A mutant, termed *large lesion mimic 1* (*llm1*), was isolated with large reddish‐brown lesions on leaves from the seedling stage to the ripening stage. After crossing *llm1 spl30‐1* with a wild‐type plant, we observed that F_2_ offsprings carrying the wild‐type *SPL30* allele (*llm1 SPL30*) also exhibited a similar large lesion phenotype as *llm1 spl30‐1*. Therefore, we concluded that *llm1* is a mutant allele in a separate locus/loci (Figure S10A).

**Figure 7:**
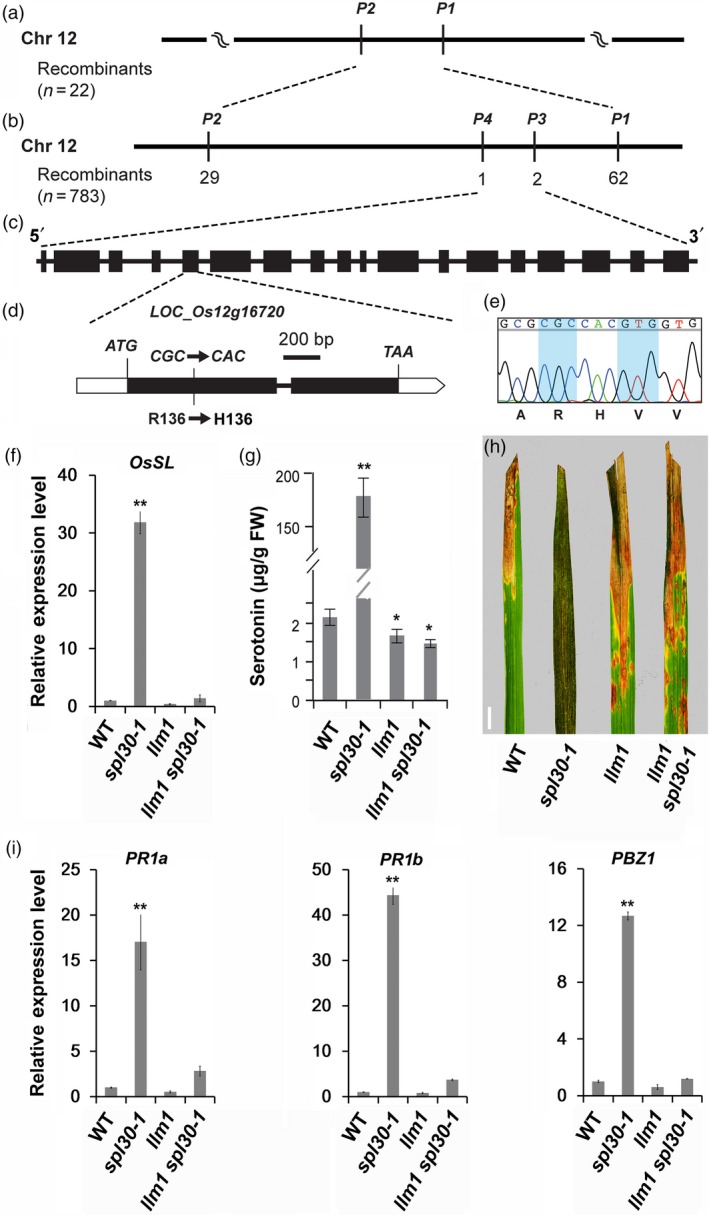
Molecular characteristics of *LLM1*. (a) *LLM1* was preliminarily mapped between markers P1 and P2 on chromosome 12; (b) Fine mapping of *LLM1*. The *LLM1* locus was mapped to a 119‐kb region between P3 and P4 markers; (c) 18 open reading frames (ORFs) were found in the region; (d, e) Sequencing result of those ORFs revealed a single nucleotide transition of G‐to‐A in the first exon of *LOC_Os12g16720*. (f) Relative expression levels of *SL* in wild‐type, *spl30‐1*,* llm1* and *llm1 spl30‐1* plants at heading stage. Error bars means ± SD of three independent replicates. ** represent significant difference at 0.01 level by Student's *t*‐test; (g) Serotonin contents in leave tissues of wild‐type, *spl30‐1*,* llm1* and *llm1 spl30‐1* plants at heading stage. FW, fresh weight. Error bars means ± SD of three independent replicates. ** represent significant difference at 0.01 level by Student's *t*‐test; (h) Leaf phenotype of wild‐type, *spl30‐1*,* llm1* and *llm1 spl30‐1* plants (from left to right) after inoculation of plant leaves with bacterial blight pathogen PXO99^A;^ bar = 1 cm; (i) Relative expression levels of *PR1a*,*PR1b* and *PBZ1* in wild‐type, *spl30‐1*,* llm1* and *llm1 spl30‐1* plants at heading stage. Error bars means ± SD of three independent replicates. ** represent significant difference at 0.01 level by Student's *t*‐test.

To map the *LLM1* locus, we crossed the *llm1* mutant with an *indica* rice variety 93–11 and collected 783 F_2_ plants exhibiting the *llm1* phenotype. Using 183 SSR and 41 STS markers that are evenly distributed on 12 chromosomes in rice, we preliminarily mapped *LLM1* between two markers P1 and P2 on chromosome 12 with 22 F_2_ individuals with the *llm1* phenotype. Further fine mapping analysis on 783 F_2_ individuals narrowed the locus within a 119‐kb region between P3 and P4 markers. According to the RGAP, 18 ORFs were found in the region. Sequencing of genomic fragments of all 18 annotated genes from *llm1* revealed a single nucleotide transition of G‐to‐A in the first exon of *LOC_Os12g16720*, which caused an Arg‐to‐His substitution at the 136th amino acid (Figure 7a–e). *LOC_Os12g16720* was previously termed *OsSL* to encode a P450 monooxygenase (Fujiwara *et al*., 2010). To verify that *llm1* is a mutant allele of *OsSL*, we crossed *llm1* with *sl*, a previously identified *OsSL* mutant. All the resulting F_1_ and F_2_ individuals exhibited an *sl*‐like phenotype (Figure S10B), indicating that *llm1* is a novel mutant allele of *OsSL*.

We then examined the expression of *OsSL* in wild‐type, *spl30‐1*,* llm1* and *llm1 spl30‐1* plants by qRT‐PCR. Compared with wild‐type, transcript level of *OsSL* was up‐regulated about 30‐fold in *spl30‐1*, reduced 2.4‐fold in *llm1*, and unchanged in *llm1 spl30‐1* plants (Figure 7f). Previous studies identified that OsSL had tryptamine 5‐hydroxylase activity for catalysing the conversion of tryptamine to serotonin, a secondary metabolite involved in strength control of cell wall thus acting as a mechanical barrier against pathogen attacks in plants (Fujiwara *et al*., 2010; Ishihara *et al*., 2008). With high‐performance liquid chromatography (HPLC) analysis, serotonin levels were detected to increase 71‐fold in *spl30‐1* and decrease 1.5‐fold in both *llm1* and *llm1 spl30‐1* plants (Figure 7g). Therefore, *SPL30* is epistatic to *LLM1/OsSL* in the serotonin metabolic pathway.

We further examined the epistatic relationship between SPL30 and OsSL by comparing the pathogen response of *spl30‐1*,* llm1* and *llm1 spl30‐1*. At 16 DPI with bacterial blight strain PXO99^A^, we detected that the leaf lesion lengths in *spl30‐1* were significantly shorter, and those in *llm1* or in *llm1 spl30‐1* plants were significantly longer than the ones observed in wild‐type plants (Figure 7h). The transcript levels of *PR1a*,* PR1b* and *PBZ1*, which were dramatically up‐regulated in *spl30‐1*, were significantly reduced or restored to the wild‐type level in the *llm1 spl30‐1* mutant (Figure 7i). These results demonstrated that *SPL30* and *LLM1/OsSL* are involved in the same pathogen response pathway and *SPL30* is epistatic to *LLM1/OsSL*.

To explore the effects of citric acid on triggering serotonin accumulation and defence response in rice, we cultivated the wild‐type plants in nutrient solution for 60 days, followed by treatment with 0 μm (control) and 500 μm citric acid for 1 week. At 16 DPI with bacterial blight strain PXO99^A^, leaf lesion lengths in 500 μm citric acid treatment was significantly shorter than the control (Figure S11A,B). Consistent with phenotype, serotonin levels were detected increased 2.3‐fold (Figure S11C), and the expression of *OsSL*,* PR1a*,* PR1b* and *PBZ1* significantly increased (Figure S11D–G) in 500 μm citric acid compared with the control. These results indicated that citric acid can enhance serotonin accumulation, and then defence response in rice.

## Discussion

### New functions of OsACL‐A2 in the regulation of PCD and innate immunity in rice

ATP‐citrate lyases has been well studied in animals in respect of tissue distribution, crystal structure, subcellular localization, enzymatic and genetic properties (Chypre *et al*., 2012). In plants, ACL consists of two subunits, ACL‐A and ACL‐B. The former is encoded by two different genes in lupin (Langlade *et al*., 2002) and sugarcane (Li *et al*., 2012), whereas three genes in Arabidopsis (Fatland *et al*., 2002); the latter is encoded by two genes in these plants. Missing of *ACL‐A* in *Micromonas pusilla* and *Ostreococcus lucimarinus* suggested that marine green algae might have adopted an alternative pathway in the acetyl‐CoA metabolism. However, we cannot rule out the possibility that this might be caused by incomplete annotation in these two genomes. Similarly, we considered that the absence of *ACL‐A* genes in *Spirodela polyrhize* was due to its incomplete genome annotation because the basal flowering plant, *Amborella trichopoda*, and all the remaining monocotyle plants analysed contained two or more *ACL‐A* loci.

In Arabidopsis, a complex phenotype has been observed in mutants with reduced ACL activity, such as miniaturized organs, smaller cells, reduced cuticular wax deposition, aberrant plastid morphology, hyperaccumulation of anthocyanin and stress‐related mRNAs in vegetative tissue (Fatland *et al*., 2005). Like Arabidopsis, the rice ACL‐A subunit is also encoded by three genes, *OsACL‐A1* (*LOC_Os11g47330*), *OsACL‐A2* (*LOC_Os12g37870*) and *OsACL‐A3* (*LOC_Os11g47120*). Although sequence alignment of amino acids showed more than 90% identities among OsACL‐A1, OsACL‐A2 and OsACL‐A3 (Figure S12), phylogenetic analysis suggested a subfunctionaliztion of OsACL‐A2 (Figure 2h). Consistently in this study, we discovered a novel function of *OsACL‐A2* in rice cell death and immune responses. The reduction in OsACL‐A2 abundance by a point mutation N343Y or by a premature stop codon resulted in evident phenotypes with small lesion mimic leaves and enhanced immunity to bacterial blight in rice (Figure 2d–g).

### The UPS‐mediated degradation of SPL30^N343Y^

In our study, the amino acid substitution (N343Y) in OsACL‐A2 located in Citrate_bind domain, which was conserved among different species (Figure S4 and Data S1). Therefore, it was suggested crucial in biological function of OsACL‐A2. In plants, the ubiquitin‐26S proteasome system is the primary machinery that targets the specific degradation of numerous intracellular proteins (Hua and Vierstra, 2011; Vierstra, 2009; Zeng *et al*., 2006). Here, the post‐translational degradation process of SPL30^N343Y^ was successfully reconstituted in a *N. benthamiana* heterologous expression system. Our results demonstrated that the point mutation N343Y enhanced a post‐translational degradation of SPL30 via a UPS‐dependent manner (Figure 4). The UPS‐mediated protein degradation often interacts with other post‐translational modifications, such as phosphorylation (Collins and Goldberg, 2017; Etlinger *et al*., 1993). Since tyrosine is one of the three major amino acids targeted for phosphorylation, we speculated that the N343Y mutation might trigger a phosphorylation‐enhanced ubiquitination.

### Potential pathways for OsACL‐A2‐mediated innate immunity in rice

Catalysed by *OsSL* (Fujiwara *et al*., 2010), serotonin has been reported to play a critical role in disease resistance (Hayashi *et al*., 2016; Jin *et al*., 2015). It served as a substrate for peroxidase in the presence of hydrogen peroxide to form a complex mixture of oligomerics that function as a physical barrier against the spread of pathogen infections in infected rice leaves (Ishihara *et al*., 2011). Our genetic analysis showed that *SPL30* is epistatic to *OsSL* in the serotonin metabolic pathway (Figure 7). The dramatically increased expression of *OsSL* in the *spl30‐1* mutant contributed to increment of serotonin contents (Figure 7f,g), which was consistent with Fujiwara *et al*. (2010) reports about serotonin synthesis catalysed by OsSL. Treatment of the *sl* mutant with serotonin enhanced its resistance to fungal by activating the expression of some pathogenesis‐related genes (Fujiwara *et al*., 2010; Hayashi *et al*., 2016). In the study, treatment of the wild‐type with citric acid enhanced its resistance to bacterial blight strain by enhancing the expression of *OsSL* and some pathogenesis‐related genes, and also serotonin contents (Figure S11). These results suggested that production of citric acid may indirectly stimulate *OsSL* expression, and then elevate defence responses in *spl30* mutants mediated by the serotonin metabolic pathway.

In order to reveal the signal pathway of *SPL30* in regulation of disease resistance, we analysed transcriptional profiles of *spl30‐1*,* spl30‐2* and wild‐type using RNA‐seq. In respect of biological process, the highest percentage of the DEGs were found up‐regulated in secondary metabolic process in both *spl30‐1* and *spl30‐2* plants (Figure S9 and Table S2), indicating the defence‐related process was enhanced in *spl30* mutants. Because disease resistance is often costly and reduces plant fitness (Deng *et al*., 2017), it is likely the main reason for yield penalty of the mutants with decrease in plant height, productive panicle number and panicle length relative to wild‐type (Figure 1c–e).

Chitinase, hydrolytic enzymes in plants, showed *in vitro* antifungal activity (Karmakar *et al*., 2016) and many *OsWRKY* transcription factor were also involved in defence mechanisms in rice (Peng *et al*., 2016). Pathogenesis‐related genes were up‐regulated by enhanced expression of several *OsWRKYs*, including *OsWRKY77* (Jimmy and Babu, 2015), a transcription factor reported to play important roles in plant pathogen responses by activating the salicylic acid (SA) pathway (Lan *et al*., 2013). Consistent with the results of RNA‐seq, transcriptional expression of two chitinase genes (*CHIT7* and *CHIT8*) and two WRKY genes (*OsWRKY77* and *OsWRKY79*) were confirmed elevated significantly by real‐time PCR in *spl30‐1* and *spl30‐2* compared to wild‐type (Figure S9C).

SA, a plant hormone, and ROS levels were highly correlated, and mutually induced each other's accumulation during plant defence responses (Rao *et al*., 1997). Phenylalanine ammonia‐lyase (PAL), the key enzyme for biosynthesis of SA in plants, contributed to responses to biotic and abiotic stress, and acted as a positive regulator of SA‐dependent defence against pathogens (Kim and Hwang, 2014; Tonnessen *et al*., 2015). Here, SA content was detected increased significantly in *spl30‐1* and *spl30‐2* mutants relative to wild‐type (Figure S13A). Coincidentally, transcriptional expression of *OsPAL4* was up‐regulated significantly in *spl30‐1* and *spl30‐2* (Figure S13B). The result indicated that disease resistance of *spl30* mutants were probably generated from the activation of the SA‐mediated defence pathway.

Based on the analyses described above, we hypothesized OsACL‐A2 may act as a negative regulatory factor to activate the *OsSL*‐mediated serotonin biosynthetic pathway and the SA‐mediated pathway, lead to activation of innate immunity.

## Experimental procedures

### Plant materials and growth conditions

The rice LMM, *spl30‐1*, was isolated from *O. sativa* ssp. *japonica* cultivar Nipponbare treated with ethyl methanesulphonate (EMS). An F_2_ mapping population was generated from a cross between *spl30‐1* and an *indica* variety NJ06. All plants were cultivated in the fields in Fuyang (FY, Zhejiang province, 119°95′E, 30°05′N) and Lingshui (LS, Hainan province, 110°02′E, 18°48′N) during rice growing season.

### Map‐based cloning and complementation test

For map‐based cloning of *SPL30*, 659 F_2_ plants with mutant‐like phenotype were generated from the cross of *spl30‐1* with *indica* rice cultivar NJ06. Gene prediction within 15.7‐kb fine mapped region on chromosome 12 was performed using the publicly available rice database, Rice Genome Annotation Project (http://rice.plantbiology.msu.edu/index.shtml).

A 7539‐bp genomic DNA fragment from Nipponbare containing the entire *SPL30* coding region, 2341‐bp promoter sequence and 1146‐bp downstream region was amplified with high‐fidelity enzyme KOD Plus (Toyobo, Tokyo, Japan) and inserted into the binary vector pCAMBIA1300. The recombinant vector was introduced into calli of the *spl30‐1* mutant via *Agrobacterium*‐mediated transformation (Toki *et al*., 2006). CRISPR/Cas9 vector was constructed according to the method of Ma *et al*. (2015). The primer sequences used are listed in Table S1.

### Phylogenetic analysis

The full set of ATP‐grasp 2 and Citrate bind seed sequences were retrieved from Pfam (Version 31, http://pfam.xfam.org) and used as query to search the predicted proteomes from 20 species (Figure 4) by BLASTp (Altschul *et al*., 1990). The presence of ATP‐grasp 2 and Citrate bind domains in a protein sequence was further annotated by HMMER3 (http://hmmer.org) against the Pfam‐A profile HMM database (Version 31, *e*‐value cutoff = 1). Sequences containing both ATP‐grasp 2 and Citrate bind functional domains were selected as SPL30 homologues from each plant species and their phylogenetic relationship was inferred using a maximum likelihood method implemented in RAxML (Version 8.1; Stamatakis, 2014) with the PROTGAMMAJTT substitution model, and the statistic significance was evaluated with 1000 bootstrap replicates using a rapid bootstrap analysis.

### Spectrophotometric assay for determination of ACL activity

Total ACL activity was determined with protocols reported previously by Fatland *et al*. (2002). The assay detects ACL‐catalysed generation of oxaloacetate by coupling the oxidation of NADH catalysed by malate dehydrogenase. The oxidation of NADH was monitored by the change in A_340_. And ACL activity was calculated using the extinction coefficient of NADH (6.22/mm/cm). The ACL assay was conducted in total volume of 1 mL, containing 200 μL of protein extracts, 20 mm MgCl_2_, 200 mm Tris‐HCl, pH 8.4, 10 mm ATP, 1 mm DTT, 10 mm citrate, 0.2 mm CoA, 0.1 mm NADH and six units of malate dehydrogenase.

### Determination of citrate and SA by HPLC

Citrate was extracted from leaf samples as published method (Phillips and Jennings, 1976) with some modifications. The 0.1 g leaf tissue at the heading stage was sampled and homogenized with 1 mL of cold acidified 80% ethanol. The homogenates were centrifuged at 10 000 *g* for 10 min and the supernatant was filtered through a syringe with a 0.22 μm PVDF membrane (Millipore, Boston, MA). The samples were analysed with HPLC using an Aminex 87H column. The mobile phase was a 0.01 N H_2_SO_4_ run at 40 °C, and a UV wavelength of 210 nm was used for detection. SA was determined with protocol reported previously by Zheng *et al*. (2015).

### Quantitative RT‐PCR

Total RNA was extracted from roots, leaves, leaf sheaths, culms and panicles at the heading stage using TRIzol reagent (Invitrogen, Shanghai, China). The extracted RNA was reverse transcribed using a SuperScriptII with gDNA remover (Invitrogen). The rice *Actin1* gene was used as an internal control. Expression values are the means of three biological repeats. The Student's *t*‐test was used for statistical analysis. Gene‐specific primers are also listed in Table S1.

### Western blot analysis

Total protein was extracted referring to a previous report (Rubio *et al*., 2005). Total protein was separated by 12% SDS‐PAGE and Western blot was performed according to a published protocol (Kurien and Scofield, 2006).

### *In vivo* SPL30^N343Y^ degradation assay

*Agrobacterium* tumefaciens containing flag‐SPL30 and flag‐ SPL30^N343Y^ together with GFP were co‐transformed into tobacco (*N. benthamiana*) leaf epidermal cells. GFP was used as an internal control to normalize the efficiency of both transformation and protein expression. For the suppression of 26S proteasome activity, the inhibitor MG132 (50 μm) or DMSO was added to the tobacco leaf epidermal cells at 48 h after transfection. Leaf tissues infiltrated with the same constructs and MG132/DMSO treatment 48 h later were mixed after being ground in liquid nitrogen and split for both protein stability and transcription analyses. Protein levels were analysed via immunoblotting with anti‐flag and anti‐GFP antibodies. *In vivo* assay for ubiquitination of SPL30 and SPL30^N343Y^ proteins was performed based on the method of Liu *et al*. (2010). The plasmid containing SPL30 or SPL30^N343Y^ fused downstream of GFP was constructed and transformed into *Agrobacterium* EHA105 cells, and then transfected into tobacco leaf epidermal cells. After 45 h, total proteins were extracted and immunoprecipitated with 25 μL GFP‐Trap‐A and then shaked at 4 °C for 2 h. The agarose beads were recovered by centrifugation at 800 rpm for 2 min and washed with cold PBS three times. The immuno‐complex was probed with anti‐Ub and anti‐GFP antibodies, respectively, via Western blot.

### Histochemical GUS assay

The promoter of *SPL30* (2341‐bp upstream of ATG) was amplified from genomic DNA of Nipponbare and inserted in‐frame into the binary vector pCAMBIA1305 with a GUS reporter gene. The recombinant vector was then introduced into calli of Nipponbare to generate transgenic plants. Different tissues of transgenic plants, involving roots, leaves, leaf sheaths, culms and panicles were used for GUS assay performed based on method of Jefferson *et al*. (1987) and Ren *et al*. (2018).

### Subcellular localization of OsACL‐A2

The cDNA sequence of *OsACL‐A2* amplified from Nipponbare was introduced into the C‐terminus of GFP with CaMV 35S promoter. The recombinant vector was then transformed into rice protoplasts and tobacco (*N. benthamiana*) leaf epidermal cells with the protocol described by Ruan *et al*. (2017).

### DAB staining, H_2_O_2_ and MDA determination and CAT activity detection

DAB staining was used to detect the accumulation of ROS based on previous study (Thordal‐Christensen *et al*., 1997). The H_2_O_2_ and MDA contents, and CAT activity were determined according to method reported by Moradi and Ismail (2007).

### TUNEL assay

Leaf tissues from the wild‐type and *spl30‐1* plants at the heading stage were isolated and exposed to TUNEL assay as described by Huang and Zhou (2007).

### RNA‐seq analysis

Total RNA from frozen leaf tissue of wild‐type (WT), *spl30‐1* and *spl30‐2* was extracted using the TRIzol Reagent according to the manufacturer's instructions (Invitrogen). The quality of total RNAs was checked using a 2100 Bioanalyzer (Agilent Technologies, Berlin, Germany). After digestion at 37 °C with DNaseI (Takara, Tokyo, Japan) for 30 min, the mRNA was purified from total RNA using the Dynabeads® Oligo (dT)25 (Life, Cleveland, OH). Construction of cDNA library was carried out following manufacturers’ instructions of NEBNext® Ultra™ RNA Library Prep Kit for Illumina (NEB, Beijing, China) and the quality and quantity of it was assessed using the Qubit™ (Thermo Fisher, Waltham, MA), eletrophoresis with 2% gel and Highsensitivity DNA chip. The 10 ng cDNA library was cluster generated with TruSeq PE Cluster Kit (Illumina, San Diego, CA) in cBot, and then paired‐end sequenced with Illumina Hiseq™2500.

RNA‐seq data were first processed for quality control by Trimmomatic (Bolger *et al*., 2014), and aligned to the rice genome via TOPHAT2 (Kim *et al*., 2013). HTSeq (http://www-huber.embl.de/users/anders/HTSeq/) programme was then applied to count an absolute expression level (counts) for each locus based on accepted hits. The list of DEGs was identified by edgeR (Robinson *et al*., 2010) with FDR < 0.05 and |log_2_(Fold change)| > 1 using normalized expression values among all samples. Gene Ontology (GO) enrichment analysis of DEGs was implemented by GOseq (Young *et al*., 2010).

### Inoculation with bacterial blight pathogen

*Xanthomonas oryzae* pv. *oryzae* strain PXO99^A^ and PXO341 were cultured on pressure‐sensitive adhesive medium. The flag leaves at the heading stage of the wild‐type, *spl30‐1*,* spl30‐2* and complementation plants were inoculated with PXO99^A^ and PXO341 suspensions with 10 independent individuals per line using the leaf clipping protocol (Manosalva *et al*., 2011). Sixteen days after inoculation, lesion lengths on inoculated leaves were measured.

### Detection of serotonin content

Serotonin content was detected as published method (Jin *et al*., 2015). 200 mg leaf tissue at the heading stage was ground with liquid nitrogen into a powder and soaked in 1 mL 100% methanol. The homogenates were centrifuged at 12 000 ***g*** for 30 min and the supernatant was filtered through a syringe with a 0.22 μm PVDF membrane (Millipore). Then the filtrate was evaporated to dryness under refrigerating vacuum pump and dissolved in 500 μL 50% methanol. The samples were separated on an XTerra RP C18 column (250 × 4.6 mm, 5 μm, Waters) with an isocratic elution of 50% methanol in water containing 0.3% trifluoroacetic acid at a flow rate of 0.4 mL/min. A UV wavelength of 280 nm was used for detection.

## Conflict of interest

The authors have declared no conflict of interest.

## Author contributions

Z, G., and Q, Q. designed research; B, R., Z, H., J, Z., B, Z., D, R., A, Z., S,Y., C, L., H, J., H, Y., J, H., L, Z.,G, C., L, S., G, Z., D, Z., G, D. and L, G. performed research; B, R., Z, H., Z, G. and Q, Q. analysed data; B, R., Z, H. and Z, G. wrote the paper.

## Supporting information


**Figure S1** (A) Phenotypes of WT and *spl30‐1* plants at the seedling stage. Scale bar = 5 cm. (B) Plant phenotype of WT, complementation lines and *spl30‐2* plants. Scale bar = 10 cm.
**Figure S2** Gene structure of *SPL30* in *spl30‐1*,* spl30‐2 and spl30‐3* plants.
**Figure S3** Sequencing chromatogram of mutated site (A) and leaf phenotype of *spl30‐3* (B). Scale bar = 1 cm.
**Figure S4** Protein sequence alignment of OsACL‐A2 and AtACL‐A genes. The *triangles* and *diamonds* below the residues show the ATP‐grasp 2 and Citrate binding sites respectively. Amino acid in white box represents the mutation site of *spl30‐1*.
**Figure S5** Expression profiles of *SPL30*. (A) Expression of *SPL30* in various organs, including root, culm, the first, second and third fully expanded leaves from the top to base of the main tiller, leaf sheath and panicle at the heading stage in wild‐type. Error bars means ± SD of three independent replicates. (B–G) GUS staining in root (B), culm (C), leaf (D), sheath (E), seed (F) and panicle (G). Scale bars = 1 cm for B, C, E, F, G; Scale bar = 2 mm for D.
**Figure S6** Subcellular localization of OsACL‐A2 protein. Transient expression of GFP (top) and OsACL‐A2‐GFP fusion (bottom) in rice protoplast (A) and epidermal cell of *Nicotiana benthamiana* leaves (B).
**Figure S7** TUNEL assay of wild‐type and *spl30‐1* leaves at heading stage with DAPI staining (top) and positive result (bottom). Scale bars = 100 μm.
**Figure S8** (A) Scatter diagram of differentially expressed genes (DEGs) with more than twofold change between *spl30‐1* and *spl30‐2* compared to WT. (B) Venn diagram showing the number of DEGs for up‐regulated and down‐regulated identified in *spl30‐1* and *spl30‐2* compared to WT.
**Figure S9** Gene ontology analysis of up‐regulated (A) and down‐regulated (B) DEGs in *spl30‐1* and *spl30‐2* compared to WT. (C) Relative expression levels of genes in WT, *spl30‐1* and *spl30‐2* plants. Error bars means ± SD of three independent replicates. ** represent significant difference at 0.01 level by Student's *t*‐test.
**Figure S10** (A) Leaf phenotype of *llm1 spl30‐1* and *llm1 SPL30*; (B) Leaf phenotype of *llm1*,* sl*, F_1_ generation of *llm1/sl*, and F_2_ generation of *llm1/sl*. Scale bars = 2 cm.
**Figure S11** (A) Phenotype of wild‐type plants 16 DPI with bacterial blight strain PXO99^A^ after treated with 0 (top) (control) and 500 μm (bottom) citric acid. (B) Lesion length after inoculation of plant leaves with bacterial blight pathogen PXO99^A^. (C) The contents of serotonin in the leaves after treated with 0 (control) and 500 μm citric acid. The expression of *OsSL* (D)*, PR1a* (E), *PR1b* (F) and *PBZ1* (G) in the leaves after treated with 0 (control) and 500 μm citric acid. Error bar means ± SD of three independent replicates. ** indicate a statistically significant difference at *P* < 0.01 by Student's *t*‐test.
**Figure S12** Protein sequence alignment of OsACL‐A1, OsACL‐A2 and OsACL‐A3.
**Figure S13** (A) Salicylic acid contents in Leaves of wild‐type, *spl30‐1* and *spl30‐2* plants at heading stage. FW, fresh weight. Error bars means ± SD of three independent replicates, ** and * indicate statistically significant difference at *P* < 0.01 and *P* < 0.05, respectively, by Student's *t*‐test. (B) Relative expression level of *OsPAL4* gene in wild‐type, *spl30‐1* and *spl30‐2* plants. Error bars means ± SD of three independent replicates. ** represents significant difference at 0.01 level by Student's *t*‐test.

**Table S1** Primers used in this study.

**Table S2** DEGs with biological process of carbohydrate metabolism, secondary metabolic process and response to biotic stimulus in *spl30‐1* and *spl30‐2* compared to WT.

**Data S1** Amino acid sequences of ACL‐A proteins in 17 plant species.
