# Supplementary material for: OsACL‐A2 negatively regulates cell death and disease resistance in rice
Source: Plant Biotechnol J. 2019 Jan 10;17(7):1344–56. doi: 10.1111/pbi.13058 (PMC6576086; doi:10.1111/pbi.13058)
Supplement: Supplementary file 1 — Figure S1 (A) Phenotypes of WT and spl30‐1 plants at the seedling stage. Scale bar = 5 cm. (B) Plant phenotype of WT, complementation lines and spl30‐2 plants. Scale bar = 10 cm. Figure S2 Gene structure of SPL30 in spl30‐1, spl30‐2 and spl30‐3 plants. Figure S3 Sequencing chromatogram of mutated site (A) and leaf phenotype of spl30‐3 (B). Scale bar = 1 cm. Figure S4 Protein sequence alignment of OsACL‐A2 and AtACL‐A genes. The triangles and diamonds below the residues show the ATP‐grasp 2 and Citrate binding sites respectively. Amino acid in white box represents the mutation site of spl30‐1. Figure S5 Expression profiles of SPL30. (A) Expression of SPL30 in various organs, including root, culm, the first, second and third fully expanded leaves from the top to base of the main tiller, leaf sheath and panicle at the heading stage in wild‐type. Error bars means ± SD of three independent replicates. (B–G) GUS staining in root (B), culm (C), leaf (D), sheath (E), seed (F) and panicle (G). Scale bars = 1 cm for B, C, E, F, G; Scale bar = 2 mm for D. Figure S6 Subcellular localization of OsACL‐A2 protein. Transient expression of GFP (top) and OsACL‐A2‐GFP fusion (bottom) in rice protoplast (A) and epidermal cell of Nicotiana benthamiana leaves (B). Figure S7 TUNEL assay of wild‐type and spl30‐1 leaves at heading stage with DAPI staining (top) and positive result (bottom). Scale bars = 100 μm. Figure S8 (A) Scatter diagram of differentially expressed genes (DEGs) with more than twofold change between spl30‐1 and spl30‐2 compared to WT. (B) Venn diagram showing the number of DEGs for up‐regulated and down‐regulated identified in spl30‐1 and spl30‐2 compared to WT. Figure S9 Gene ontology analysis of up‐regulated (A) and down‐regulated (B) DEGs in spl30‐1 and spl30‐2 compared to WT. (C) Relative expression levels of genes in WT, spl30‐1 and spl30‐2 plants. Error bars means ± SD of three independent replicates. ** represent significant difference at 0.01 level by Student's t‐ [file PBI-17-1344-s002.doc]

**
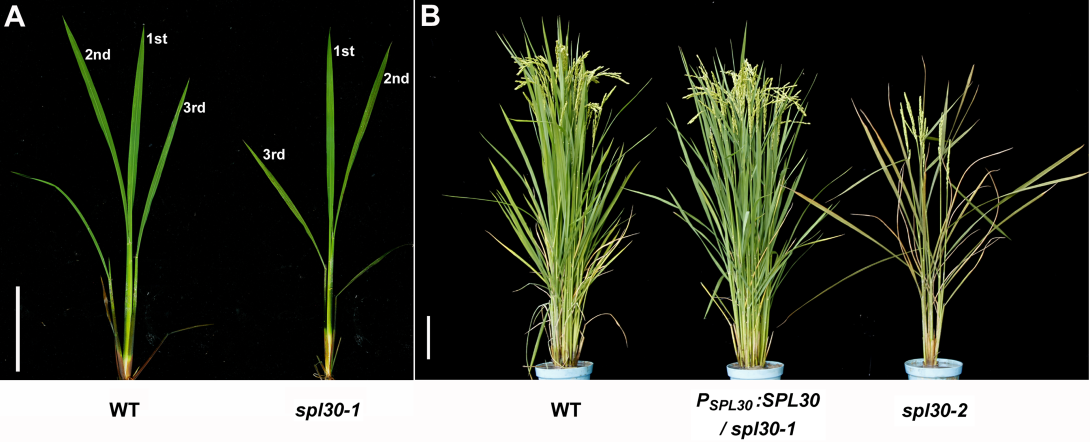
**

**Fig. S1** A. Phenotypes of WT and *spl30-1* plants at the seedling stage. Scale bar = 5 cm. B. Plant phenotype of WT, complementation lines and *spl30-2* plants. Scale bar = 10 cm.

**
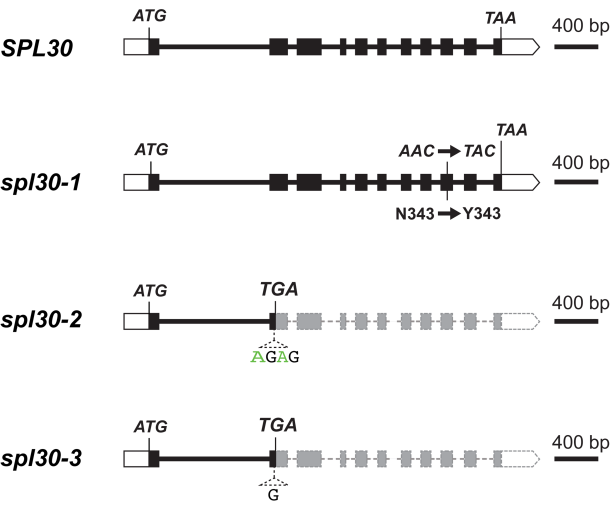
**

**Fig. S2** Gene structure of *SPL30* in *spl30-1*, *spl30-2 and spl30-3* plants.

**
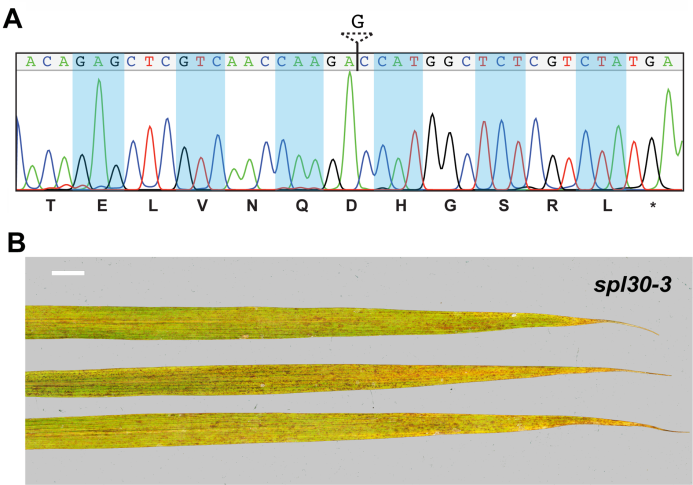
**

**Fig. S3** Sequencing chromatogram of mutated site (A) and leaf phenotype of *spl30-3* (B). Scale bar = 1 cm.

**
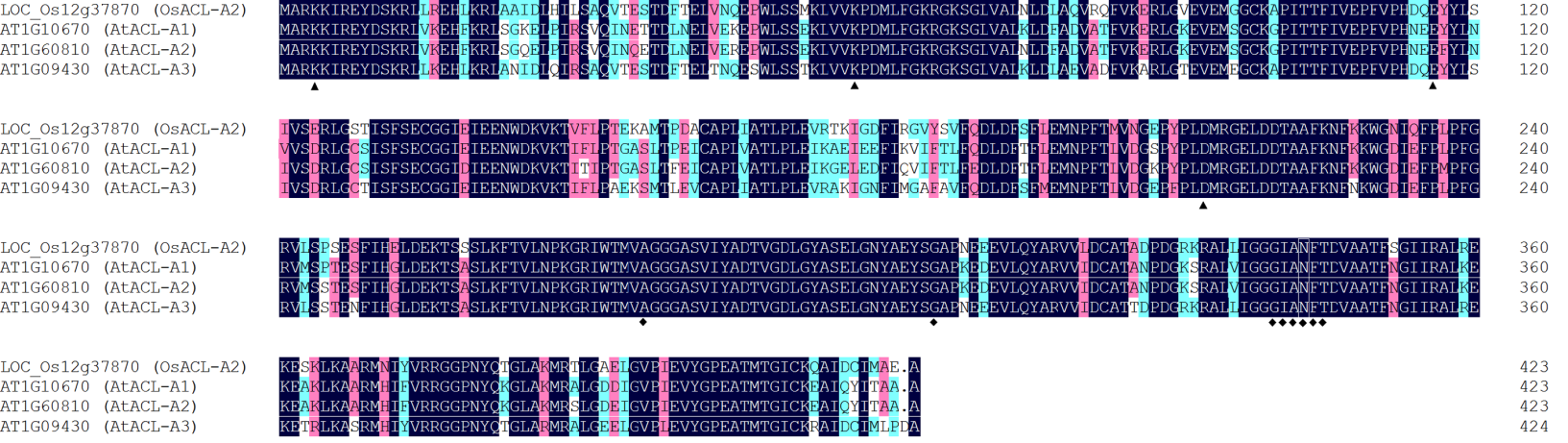
**

**Fig. S4** Protein sequence alignment of OsACL-A2 and AtACL-A genes. The *triangles* and *diamonds* below the residues show the ATP-grasp 2 and Citrate_binding sites, respectively. Amino acid in white box represents the mutation site of *spl30-1*.

**
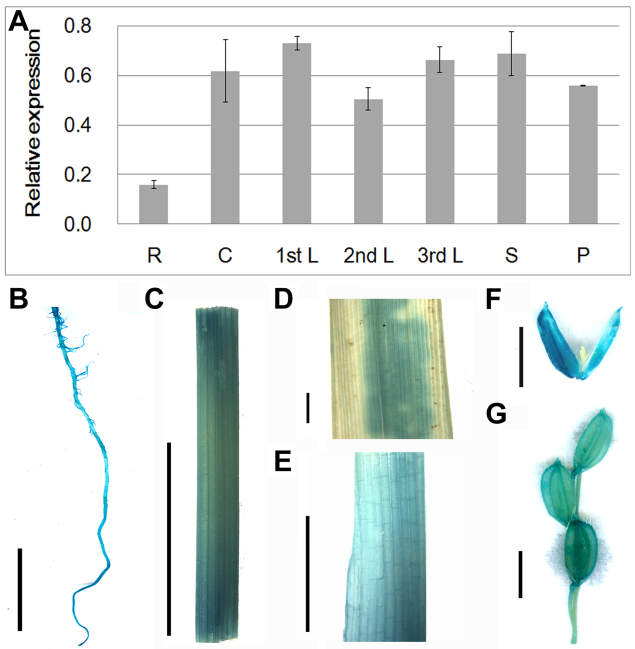
**

**Fig. S5** Expression profiles of *SPL30*. A. Expression of *SPL30* in various organs, including root, culm, the first, second and third fully expanded leaves from the top to base of the main tiller, leaf sheath and panicle at the heading stage in wild-type. Error bars means ±SD of three independent replicates. B-G. GUS staining in root (B), culm (C), leaf (D), sheath (E), seed (F) and panicle (G). Scale bars = 1 cm for B, C, E, F, G; Scale bar = 2 mm for D.

**
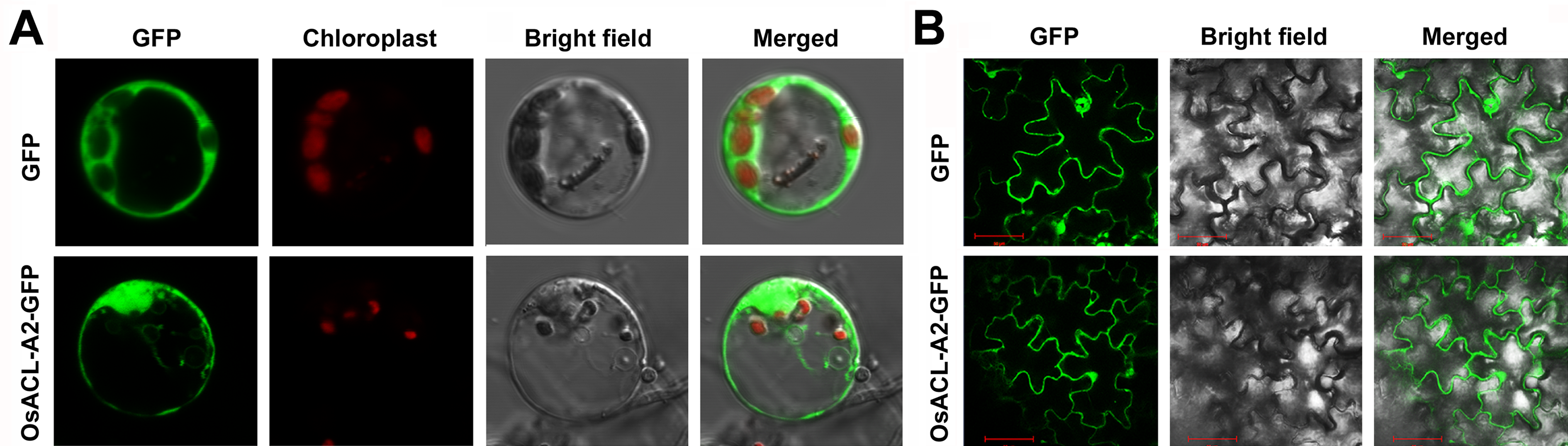
**

**Fig. S6** Subcellular localization of OsACL-A2 protein. Transient expression of GFP (top) and OsACL-A2-GFP fusion (bottom) in rice protoplast (A) and epidermal cell of *N. benthamiana* leaves (B).

**
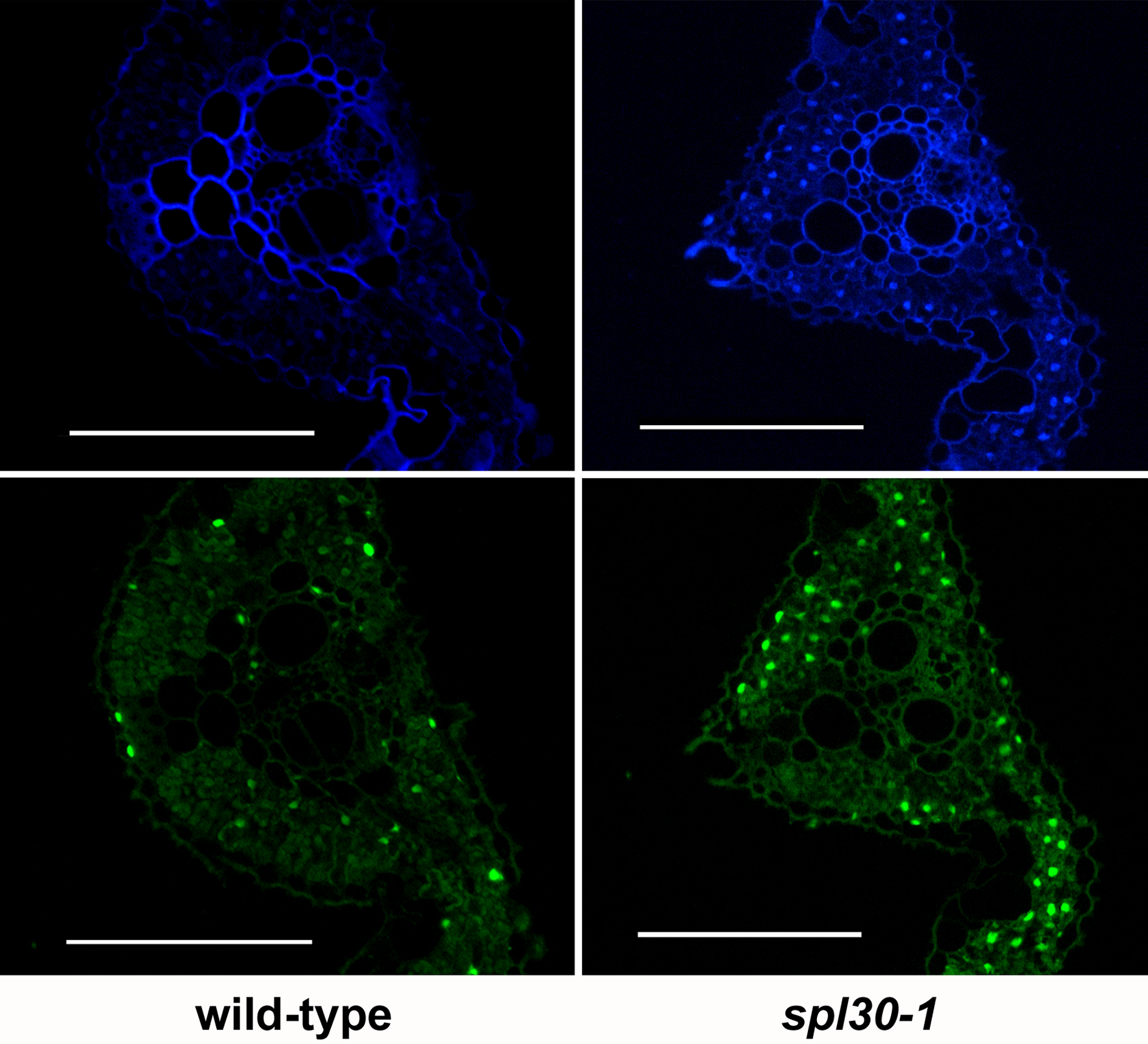
**

**Fig. S7** TUNEL assay of wild-type and *spl30-1* leaves at heading stage with DAPI staining (top) and positive result (bottom). Scale bars = 100 μm.

**
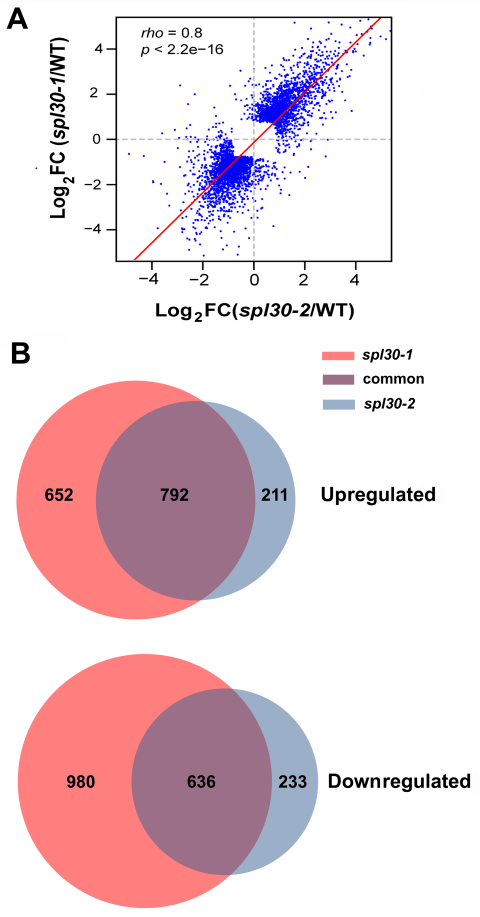
**

**Fig. S8** A. Scatter diagram of differentially expressed genes (DEGs) with more than 2-fold change between *spl30-1* and *spl30-2* compared to WT. B. Venn diagram showing the number of DEGs for upregulated and downregulated identified in *spl30-1* and *spl30-2* compared to WT.

**
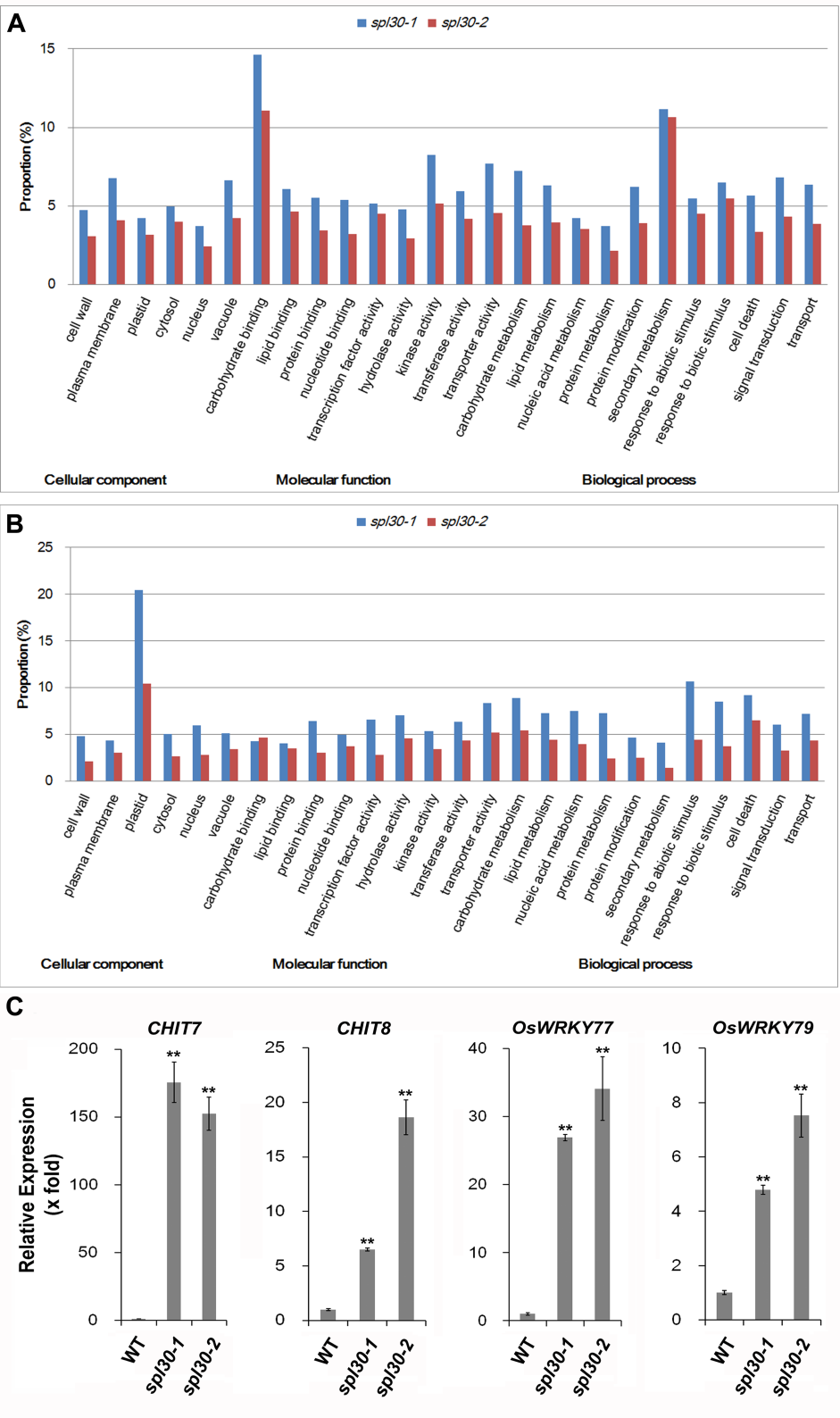
**

**Fig. S9** Gene ontology analysis of upregulated (A) and downregulated (B) DEGs in *spl30-1* and *spl30-2* compared to WT. C. Relative expression levels of genes in WT, *spl30-1* and *spl30-2* plants. Error bars means ±SD of three independent replicates. ** represent significant difference at 0.01 level by student *t*-test.

**
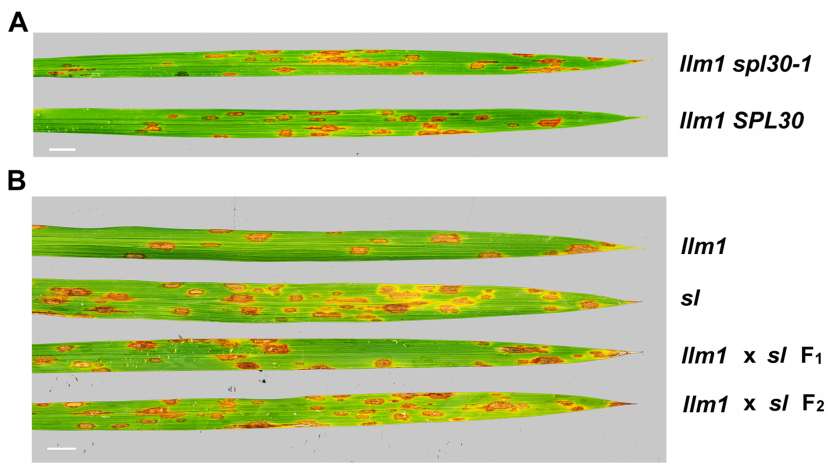
**

**Fig. S10** A. Leaf phenotype of *llm1 spl30-1* and *llm1 SPL30*; B. Leaf phenotype of *llm1*, *sl*, F_1_ generation of *llm1/sl*, and F_2_ generation of *llm1/sl*. Scale bars = 2 cm.

**
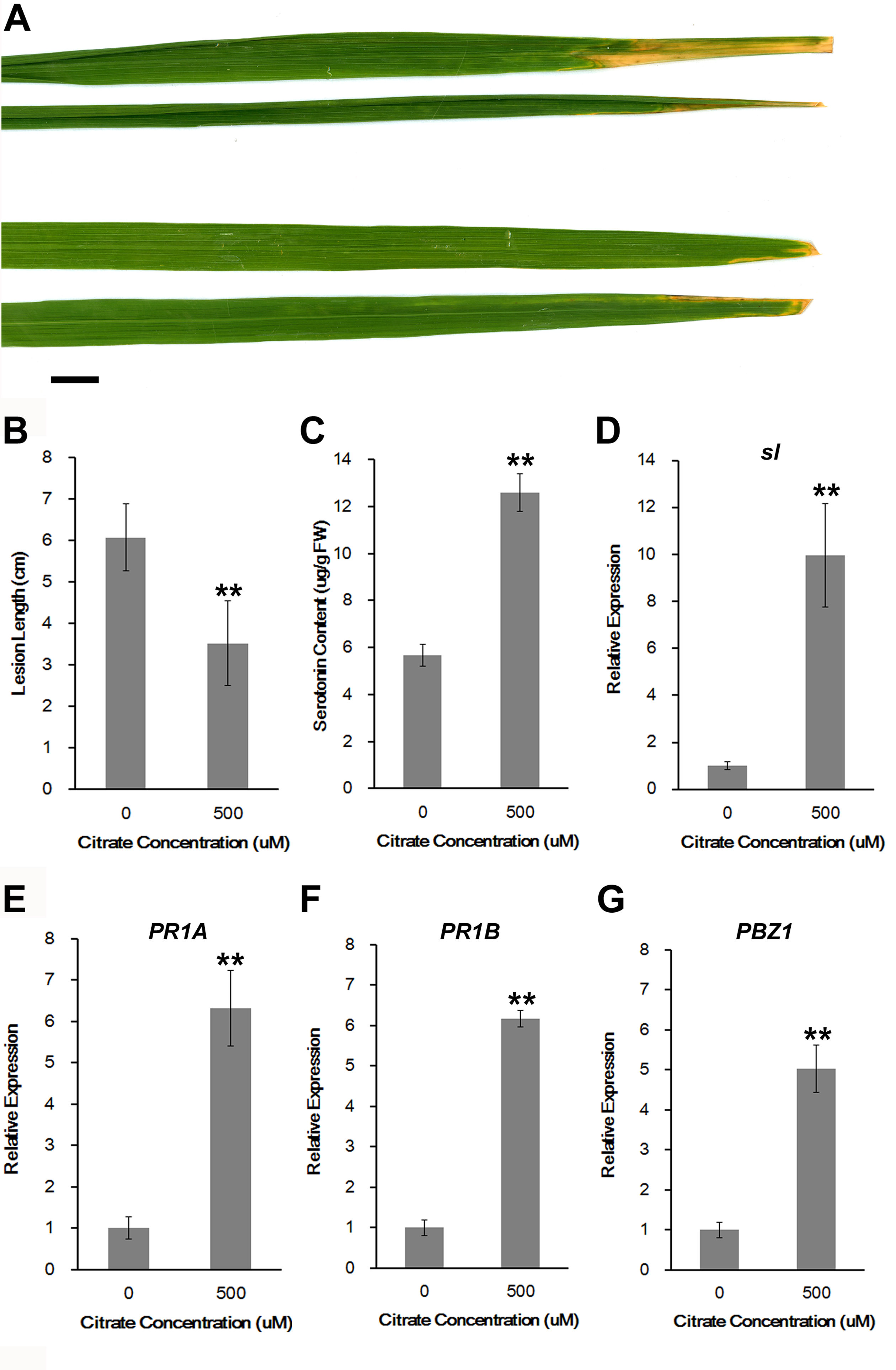
**

**Fig. S11** A. Phenotype of wild type plants 16 DPI with bacterial blight strain PXO99^A^ after treated with 0 (top) (control) and 500 μM (bottom) citric acid. B. Lesion length after inoculation of plant leaves with bacterial blight pathogen PXO99^A^. C. The contents of serotonin in the leaves after treated with 0 (control) and 500 μM citric acid. The expression of *OsSL* (D)*, PR1a* (E), *PR1b* (F) and *PBZ1* (G) in the leaves after treated with 0 (control) and 500 μM citric acid. Error bar means ±SD of three independent replicates. ** indicate a statistically significant difference at P<0.01 by student *t*-test.

**
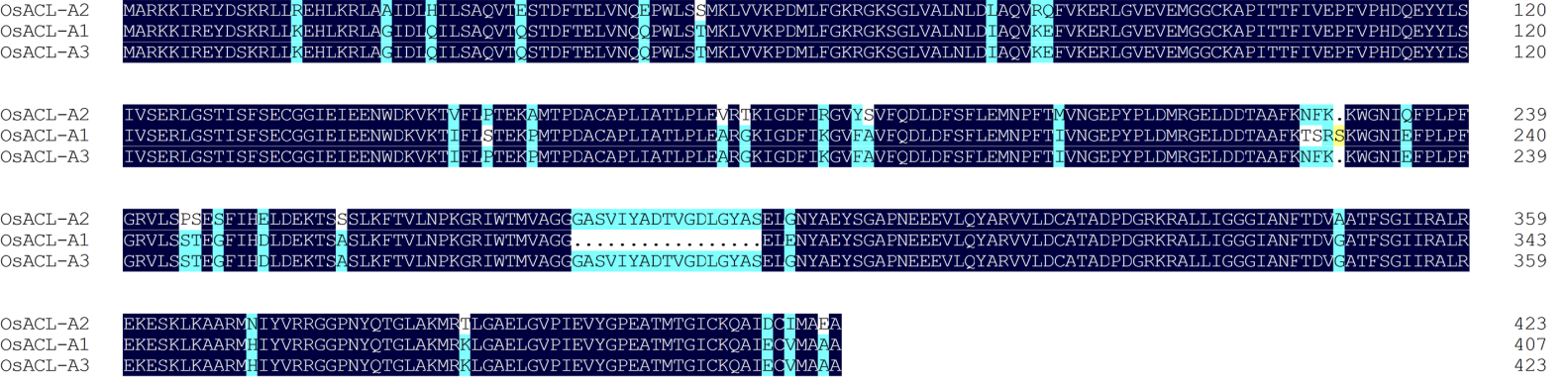
**

**Fig. S12** Protein sequence alignment of OsACL-A1, OsACL-A2 and OsACL-A3.

**
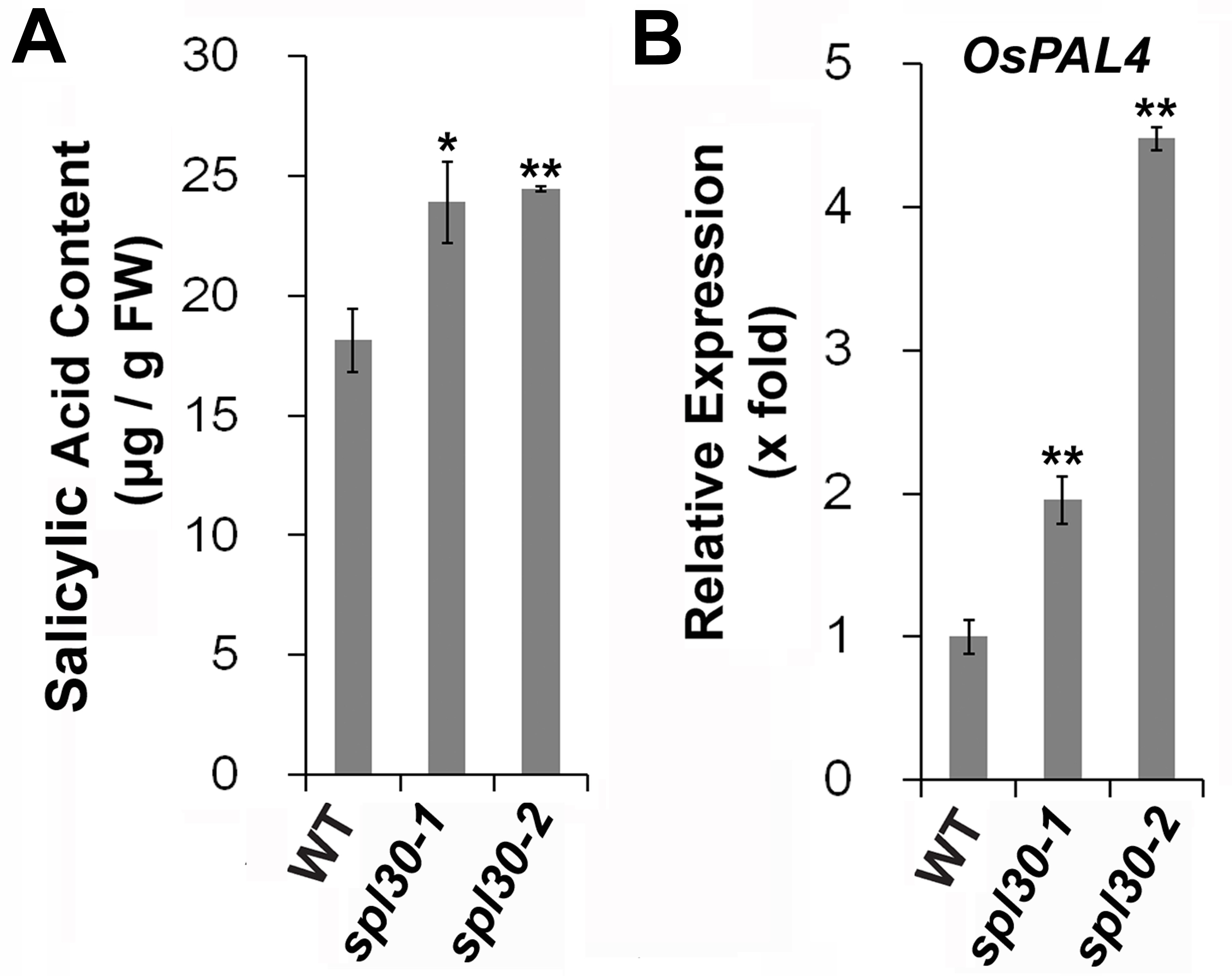
**

**Fig. S13** A. Salicylic acid contents in Leaves of wild-type, *spl30-1* and *spl30-2* plants at heading stage. FW, fresh weight. Error bars means ±SD of three independent replicates, * and ** represent significant difference at 0.05 and 0.01 level by student *t*-test, respectively. B. Relative expression level of *OsPAL4* gene in wild type, *spl30-1* and *spl30-2* plants. Error bars means ±SD of three independent replicates. ** represents significant difference at 0.01 level by student *t*-test.
