# Supplementary material for: OsACL‐A2 negatively regulates cell death and disease resistance in rice
Source: Plant Biotechnol J. 2019 Jan 10;17(7):1344–56. doi: 10.1111/pbi.13058 (PMC6576086; doi:10.1111/pbi.13058)
Supplement: Supplementary file 2 — Table S1 Primers used in this study. [file PBI-17-1344-s004.doc]

Supplemental Table 1. Primers used in this study.

| Primer | Primer sequence | Description |
| --- | --- | --- |
| R1 | F, 5'-CCTCTTAAACACACCCGAA-3' | Mapping |
|  | R, 5'-CAATGGTTGAGATTCGGAGA-3' |  |
| R3 | F, 5'-GGTGTTTTCGTCTACACTTG-3' | Mapping |
|  | R, 5'-CCTGATTCTTTGGGTAACCT-3' |  |
| R4 | F, 5'-GAGTCGTGGATGTAAAACTC-3' | Mapping |
|  | R, 5'-GCAAAGAGATGCACACATATAGTG-3' |  |
| R5 | F, 5'-AGTGGCCTAGGTAGATTGAA-3' | Mapping |
|  | R, 5'-TCACAAGGCTATCTCTCTTTG-3' |  |
| R6 | F, 5'-CAAGCCGAAGACGAAGTC-3' | Mapping |
|  | R, 5'-CAGCGAGACAGGTTACAC-3' |  |
| R7 | F, 5'-GGCGGAGGCACTTGA-3' | Mapping |
|  | R, 5'-ATTGAAGCGGACGACAC-3' |  |
| R8 | F, 5'-TCCTCCCATCTTCTCTTCTCTTCC-3' | Mapping |
|  | R, 5'-CTAGGCCTCGTCCTGAGACTGG-3' |  |
| R9 | F, 5'-ACCGTTAGATGACACAAGCAACG-3' | Mapping |
|  | R, 5'-GGTTAGCAAGACTGGAGGAGACG-3' |  |
| R10 | F, 5'-ACTTCGGCCACGACCAAGTAGG-3' | Mapping |
|  | R, 5'-CGCACATCCAGTTCATCAATTCC-3' |  |
| P1 | F, 5'-GTGAATGGTCAAGTGACTTAGGTGGC-3' | Mapping |
|  | R, 5'-ACACAACATGTTCCCTCCCATGC-3' |  |
| P2 | F, 5'-TCCTTCTCGTTTATGAACTTATGG-3' | Mapping |
|  | R, 5'-TAGAGCAAAGCAGAGCCCAG-3' |  |
| P3 | F, 5'-TGTGTCACATCGGACGTTTG-3' | Mapping |
|  | R, 5'-TGCTACCGTAACCCACATG-3' |  |
| P4 | F, 5'-CACCGAATGCAAAGCCGAG-3' | Mapping |
|  | R, 5'-GCGGCGTTGTGACCGTCGG-3' |  |
| Cas-*SPL30* | F, 5'-ggcaGCTCGTCAACCAAGAGCCA-3' | CRISPR/Cas9 construction |
|  | R, 5'-aaacTGGCTCTTGGTTGACGAGC-3' |  |
| Com-*SPL30* | F, 5'-cccggtaccTTGCAAGGATGCCACTCGAA-3' | Complementation construction |
|  | R, 5'-aaagtcgacGGCTACTGCGGTATCCCTTC-3' |  |
| Gus-*SPL30* | F, 5'-cccggtaccTTGCAAGGATGCCACTCGAA-3' | GUS construction |
|  | R, 5'-caaccatGGCTCTCTGCCTCTCTCTCTCTCTCTCTC-3' |  |
| GFP-*SPL30* | F, 5'-aaaggatccATGGCGCGGAAGAAGATCCGGGAGTACGACT-3' | Sub-cellular localization construction |
|  | R, 5'-aaaggatccTTATGCTTCAGCCATGATGCAATCAATGGC-3' |  |
| PET28a-*SPL30* | F, 5'-aaaggatccATGGCGCGGAAGAAGATCCGGGAGTACGACT-3' | Prokaryotic expression construction |
|  | R, 5'-aaactcgagTGCTTCAGCCATGATGCAATCAATGGC-3' |  |
| 870flag-*SPL30* | F, 5'-cacgggggactctagaATGGCGCGGAAGAAGATCCGG-3' | flag-SPL30 and flag-SPL30N343Y construction |
|  | R, 5'-cgggggatcctctagaTGCTTCAGCCATGATGCAATC-3' |  |
| *SPL30*-RT | F, 5'-CCTGATGGCCGTAAGAGAGC-3' | qPCR analysis |
|  | R, 5'-TCATCCGTGCAGCCTTCAAT-3' |  |
| *SGR*-RT | F, 5'-GCAATGTCGCCAAATGACG-3' | qPCR analysis |
|  | R, 5'-GCTCACCACACTCATTCCCTAAAG-3' |  |
| *OsWRKY23*-RT | F, 5'-TCCAGTTCCTCTCCCAGTTCTAA-3' | qPCR analysis |
|  | R, 5'-CACATTGTTCTCCTTTTCTTCCC-3' |  |
| *Osh36*-RT | F, 5'-TTGAGCGGTAGCACCCCATT-3' | qPCR analysis |
|  | R, 5'-GCACGGAGGCGAACGA-3' |  |
| *Osl57*-RT | F, 5'-ACCCTAAAGTAAATGAAGTC-3' | qPCR analysis |
|  | R, 5'-CCTGCTCTTGTCTTGTTA-3' |  |
| *PR1a*-RT | F, 5'-CGTGTCGGCGTGGGTGT-3' | qPCR analysis |
|  | R, 5'-GGCGAGTAGTTGCAGGTGATG-3' |  |
| *PR1b*-RT | F, 5'-TACGCCAGCCAGAGGAGC-3' | qPCR analysis |
|  | R, 5'-GCCGAACCCCAGAAGAGG-3' |  |
| *PBZ1*-RT | F, 5'-GGTGTGGGAAGCACATACAA-3' | qPCR analysis |
|  | R, 5'-GTCTCCGTCGAGTGTGACTTG-3' |  |
| *SL*-RT | F, 5'-AAGGGAGAGGAGCTTAGTGAGG-3' | qPCR analysis |
|  | R, 5'-AGCTCCAGCCTCCAGGTTACTTAG-3' |  |
| *CHIT7*-RT | F, 5'-CACCAACATCATCAACGGCG-3' | qPCR analysis |
|  | R, 5'-GGGCCTCTGGTTGTAGCAAT-3' |  |
| *CHIT8*-RT | F, 5'-GACCAACATCATCAACGGCG-3' | qPCR analysis |
|  | R, 5'-ATGTCGCAGTAGCGCTTGTA-3' |  |
| *OsWRKY77*-RT | F, 5'-TACGACGGCGTCCACAAC-3' | qPCR analysis |
|  | R, 5'-GAAGAGAGCGATCACCTCGG-3' |  |
| *OsWRKY79*-RT | F, 5'-CAGCCTCGGTTTTGTGGTCG-3' | qPCR analysis |
|  | R, 5'-TCGAACGGGAAGAAGTCATCG-3' |  |
| *OsPAL4*-RT | F, 5'-TGGCGAGGAGCTGAACAAGGTTC-3' | qPCR analysis |
|  | R, 5'-TGAGGCACTCGAGCAACGGG-3' |  |
| *Actin* | F, 5'-GGAAGTACAGTGTCTGGATTGGAG-3' | qPCR analysis |
|  | R, 5'-TCTTGGCTTAGCATTCTTGGGT-3' |  |
